# Temporal Profile of the Renal Transcriptome of HIV-1 Transgenic Mice during Disease Progression

**DOI:** 10.1371/journal.pone.0093019

**Published:** 2014-03-25

**Authors:** Ying Fan, Chengguo Wei, Wenzhen Xiao, Weijia Zhang, Niansong Wang, Peter Y. Chuang, John Cijiang He

**Affiliations:** 1 Department of Nephrology, Shanghai Jiao Tong University Affiliated Sixth People's Hospital, Shanghai, China; 2 Division of Nephrology, Department of Medicine, Icahn School of Medicine at Mount Sinai, New York, New York, United States of America; University of Kentucky, United States of America

## Abstract

Profiling of temporal changes of gene expression in the same kidney over the course of renal disease progression is challenging because repeat renal biopsies are rarely indicated in clinical practice. Here, we profiled the temporal change in renal transcriptome of HIV-1 transgenic mice (Tg26), an animal model for human HIV-associated nephropathy (HIVAN), and their littermates at three different time points (4, 8, and 12 weeks of age) representing early, middle, and late stages of renal disease by serial kidney biopsy. We analyzed both static levels of gene expression at three stages of disease and dynamic changes in gene expression between different stages. Analysis of static and dynamic changes in gene expression revealed that up-regulated genes at the early and middle stages are mostly involved in immune response and inflammation, whereas down-regulated genes mostly related to fatty acid and retinoid metabolisms. We validated the expression of a selected panel of genes that are up-regulated at the early stage (CCL2, CCL5, CXCL11, Ubd, Anxa1, and Spon1) by real-time PCR. Among these up-regulated genes, Spon1, which is a previously identified candidate gene for hypertension, was found to be up-regulated in kidney of human with diabetic nephropathy. Immunostaining of human biopsy samples demonstrated that protein expression of Spon1 was also markedly increased in kidneys of patients with both early and late HIVAN and diabetic nephropathy. Our studies suggest that analysis of both static and dynamic changes of gene expression profiles in disease progression avails another layer of information that could be utilized to gain a more comprehensive understanding of disease progression and identify potential biomarkers and drug targets.

## Introduction

Most patients with chronic kidney disease (CKD) progress to end stage renal failure (ESRD) despite medical intervention [1] [2]. One of the reasons is that biomarkers for early detection of the kidney disease are lacking. Therefore, we are unable to intervene early before irreversible damage. In order to identify early biomarkers and drug targets for progression of CKD, it is critical to understand the cellular and molecular mechanisms underlying the development and progression of disease.

Transcriptome-based approach has been widely applied for studying diabetic nephropathy (DN) [3] [4], focal segmental glomerulosclerosis [5], chronic kidney disease progression [6], and glomerular disease classification [7]. The transcriptiomic approach is one of the most promising and advanced methods for identifying biomarkers and studying disease pathogenesis. However, this approach is not without its limitations. First, access to renal biopsy samples are often limited due to the small volume of core needle sample and the relatively scarce number of routine biopsies performed in general nephrology practice. Second, most kidney biopsies are performed on patients with established disease. Hence, early changes in gene expression remain largely unknown. Third, in most cases repeat sampling of the kidney is not done if patients respond to therapy. Therefore, it is impossible to obtain a temporal change of gene profiles in patients over the entire course of the disease. Due to these factors, the clinical utility of current human transcriptomic data is limited. Some of these limitations, however, could be overcome by studying animal models of kidney disease. Here, we examined the temporal profile of gene expression over the course of disease progression by serial sampling of the kidney.

Many animal models have been used to study the pathogenesis and progression of kidney disease. However, most animal models develop only mild kidney disease without progression to renal failure, which is the case for almost all experimental models of diabetic nephropathy [8]. HIV-1 transgenic mouse model (Tg26) has been used extensively to study the pathogenesis of HIVAN because these mice develop renal disease mimicking human HIVAN [9]. Tg26 mice develop proteinuria as early as 4 weeks of age and proteinuria peaks at 8 weeks of age. Tg26 mice develop mild glomerulosclerosis (GS) at 4 weeks of age, moderate GS and mild tubulointerstitial injury at 8 weeks of age, and advanced GS and tubulointerstitial fibrosis, tubular atrophy and dilatation at 12 weeks of age [10]. Tg26 mice have rapid progression of kidney disease to renal failure and usually die from uremia between the ages of 2 to 6 months. Variability in disease progression is thought to be due to genetic penetrance [11]. Therefore, Tg26 mouse is a robust model to study the progression of kidney disease.

In the current study, we performed serial kidney biopsies in Tg26 mice and age and gender-matched control littermates at 4 weeks and 8 weeks of age and mice were sacrificed at 12 weeks of age. Gene expression profiles in the kidney cortices of Tg26 and their control littermates at these three time points were assessed by next-generation sequencing of mRNA extracted from the kidney cortex. Transcriptomic data were analyzed to identify temporal pattern of gene expression during disease progression. To determine cellular processes and genes that could be drivers of disease progression, we focused on the genes that are differentially regulated during the early stage of disease.

## Results

### Natural history of renal disease of HIV-1 transgenic, Tg26, mice

As shown in Table 1, Tg26 mice developed mild proteinuria at 4 weeks of age, moderate proteinuria at age of 8 weeks, and severe proteinuria at the age of 12 weeks, while control wild type (WT) littermates had no marked urinary albumin excretion. Kidney tissues from three Tg26 and three WT mice were obtained by open biopsies at ages of 4 and 8 weeks and when mice were sacrificed a 12 weeks of age. Tg26 mice developed mild GS without significant tubulointerstitial injury at 4 weeks of age, moderate GS with mild tubulointerstitial injury at 8 weeks of age, and severe GS and interstitial fibrosis and inflammation with tubular atrophy and dilatation at 12 weeks of age.

**Table 1:** Proteinuria and histology of Tg26 mice at three time points.

	Albuminuria/Cr ratio	% Glomerulosclerosis	% Tubular atrophy/Interstitial Fibrosis
WT (n = 3)	0	0%	0%
Tg-4wks (n = 3)	0.6; 0.8; 1.2;	8%; 14%, 20%	0%; 0%; 0%
Tg-8wks (n = 3)	1.5; 1.8; 1.9	28%; 32%; 35%	5%; 8%; 10%
Tg-12wks (n = 3)	2.5; 2.8; 3.0	52%; 56%; 68%	20%; 25%; 28%

Summary of proteinuria and kidney histology: Proteinuria was measured by determination of urinary albumin to creatinine ratio. Periodic acid-Schiff (PAS)-stained kidney sections from *Tg26* mice and the littermates were graded by a renal pathologist blinded to genotypes as described in the method. Three numbers in each cell represent the data from three individual animals.

### Analysis of static levels of gene expression profiles in Tg26 kidneys at 4, 8, and 12 weeks

**Figure 1:**
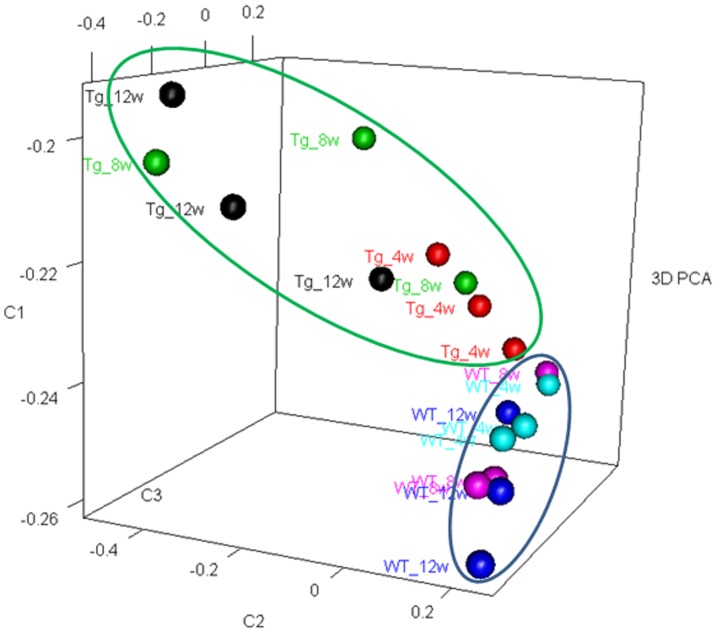
3D snapshot of PCA analysis of samples distribution based on all the genes: The Tg26 samples were well separated from WT samples. Moreover, 8

**Figure 2:**
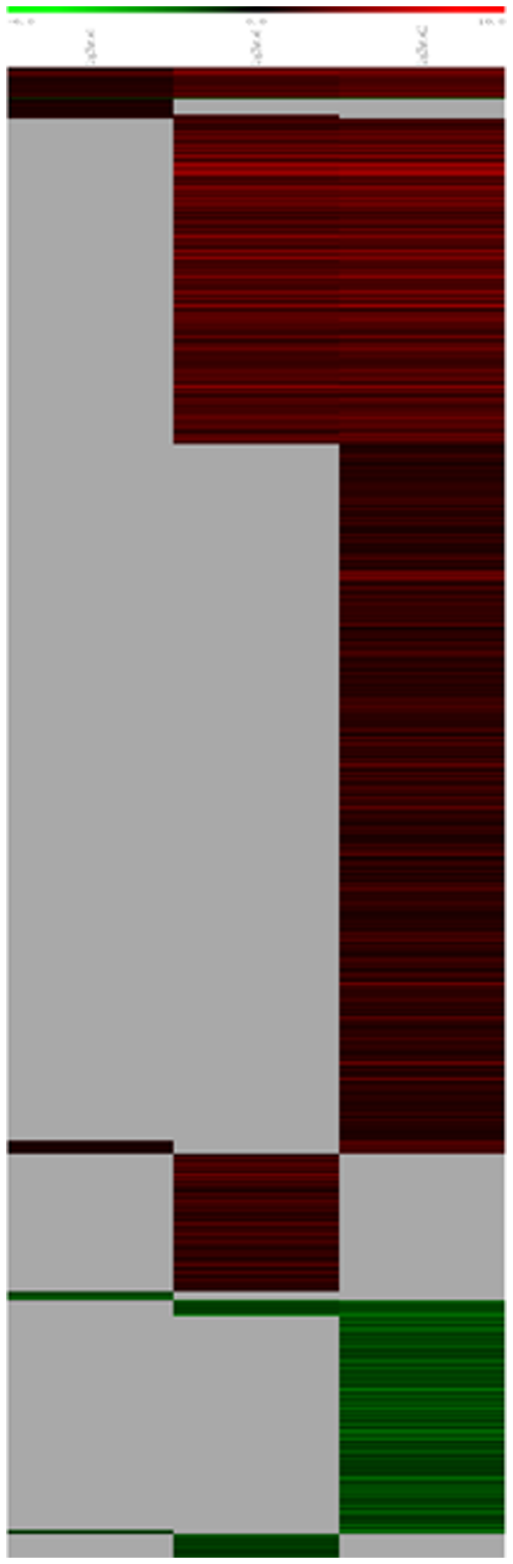
The Heat Map of gene expression profiles in the kidney of Tg26 mice at three time points: 4, 8, and 12 weeks of age when mice developed mild, moderate and severe kidney injury. The data were normalized by comparing to control littermate mice as described in the method.

**Figure 3:**
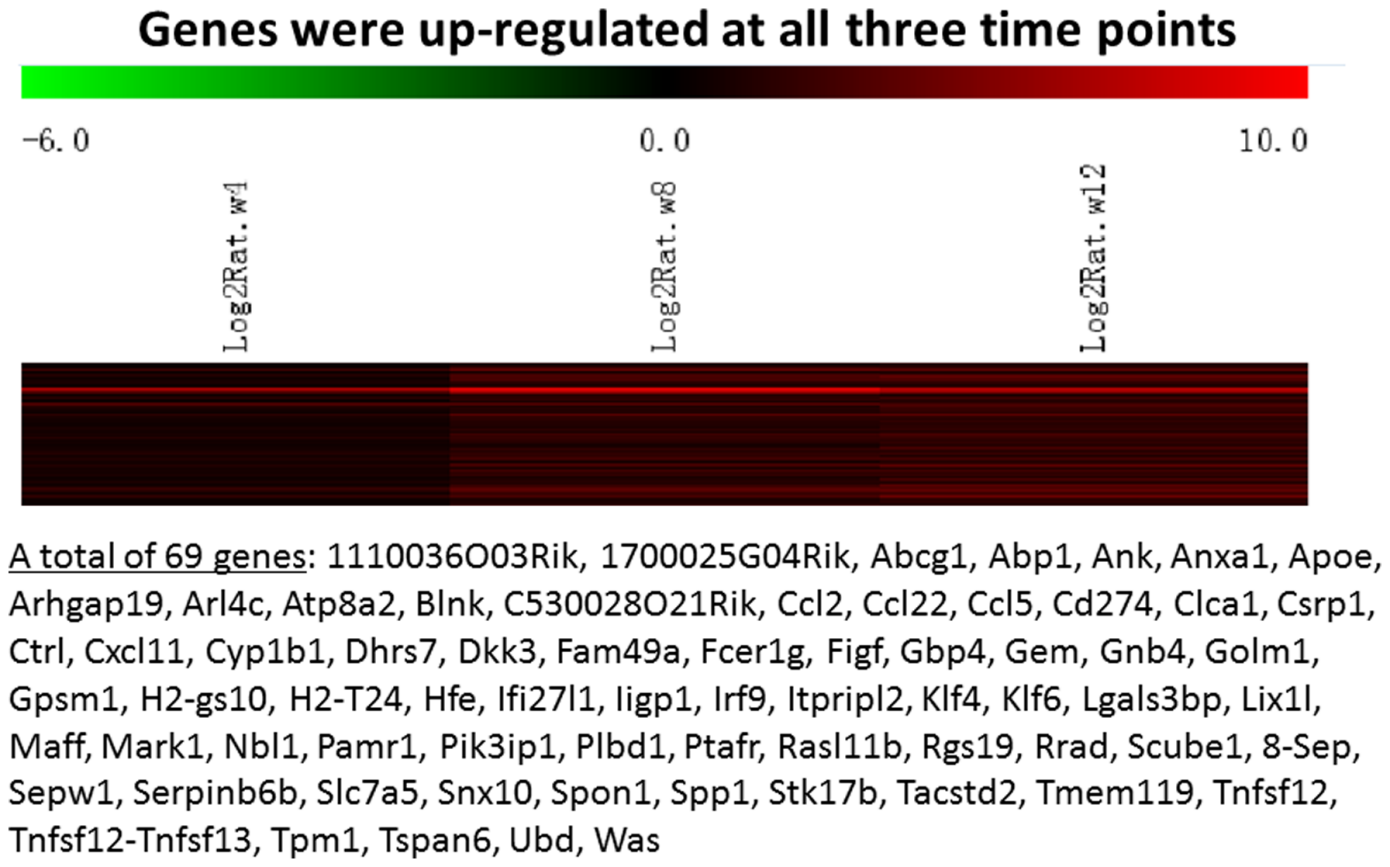
The Heat Map and the list of genes which were up-regulated at all three time points (4, 8, 12 weeks of age). There were a total of 69 genes in the list.

**Figure 4:**
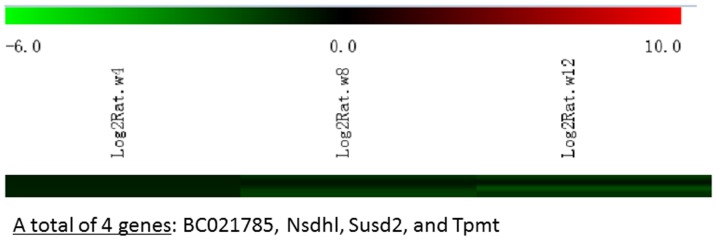
The Heat Map and the list of genes which were down-regulated at all three time points (4, 8, 12 weeks of age). There were only 4 genes in the list.

**Figure 5:**
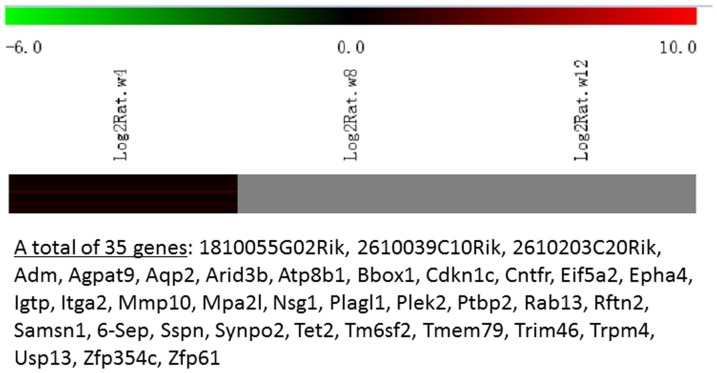
The Heat Map and the list of genes which were up-regulated at the early stage (4 weeks of age). There were a total of 35 genes in the list.

**Figure 6:**
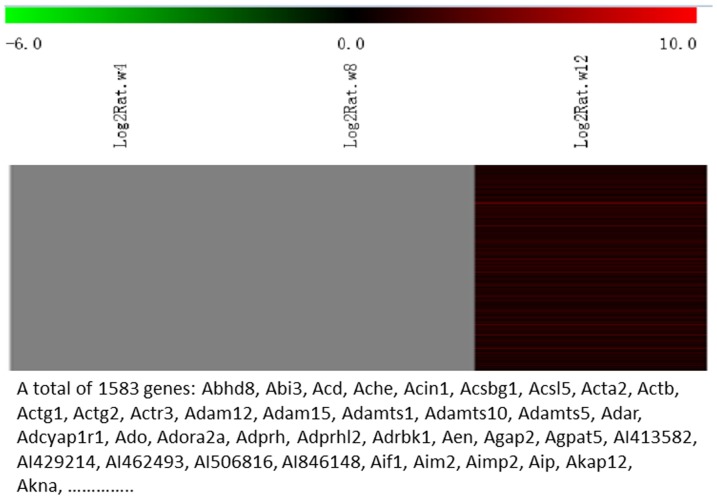
The Heat Map and the list of genes which were up-regulated at the late stage (12 weeks of age). There were a total of 1583 genes in the list.

**Figure 7:**
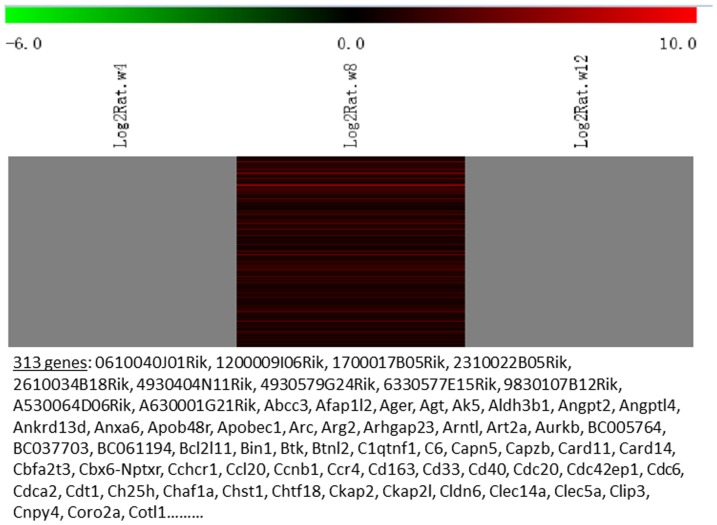
The Heat Map and the list of genes which were up-regulated transiently at the middle stage (8 weeks of age). There were a total of 313 genes in the list.

**Figure 8:**
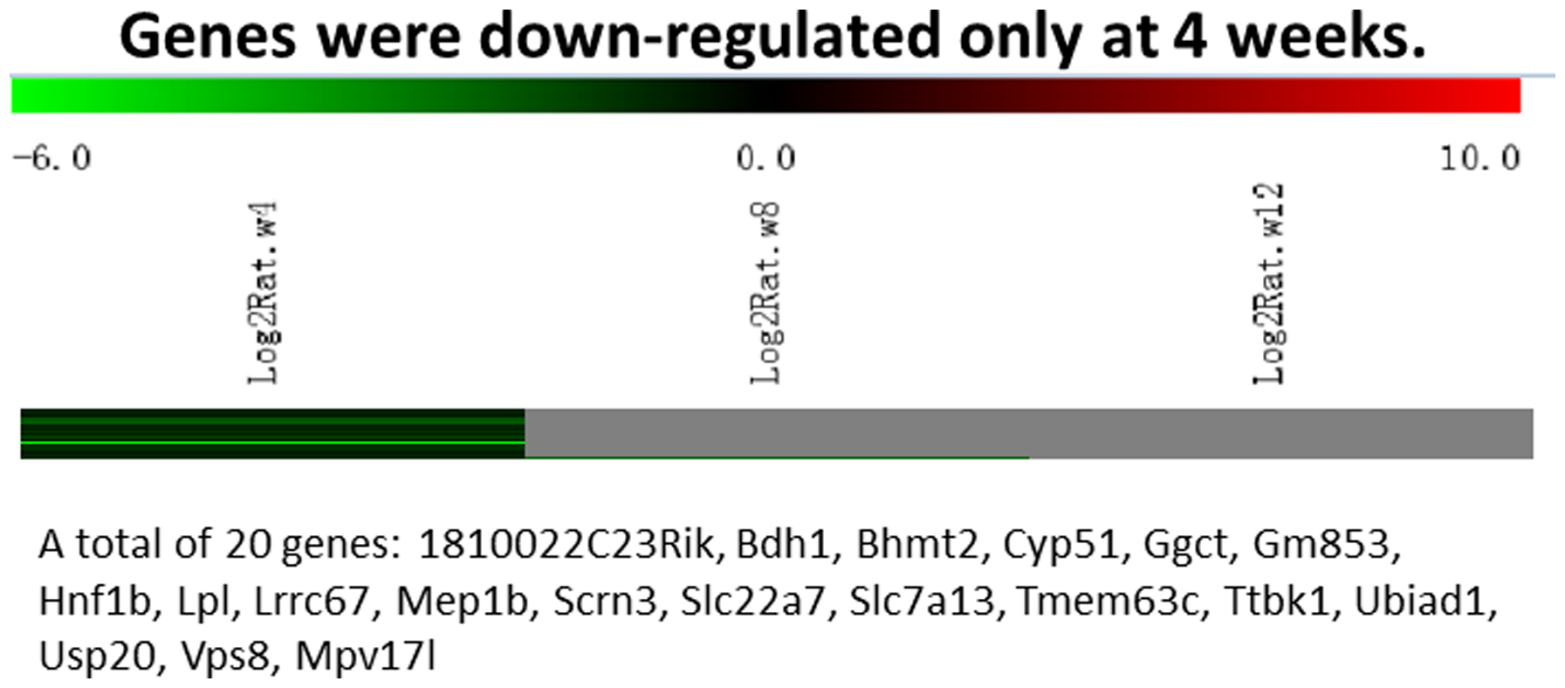
The Heat Map and the list of genes which were down-regulated at the early stage (4 weeks of age). There were a total of 20 genes in the list.

**Figure 9:**
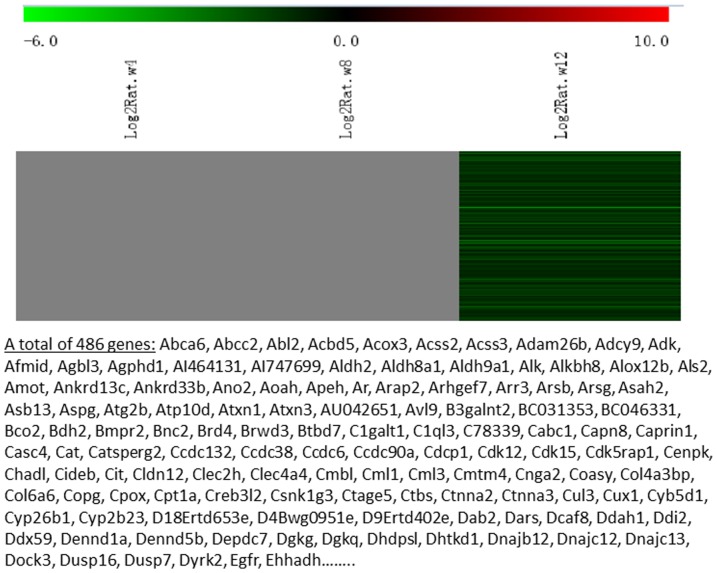
The Heat Map and the list of genes which were down-regulated at the late stage (12 weeks of age). There were a total of 486 genes in the list.

**Figure 10:**
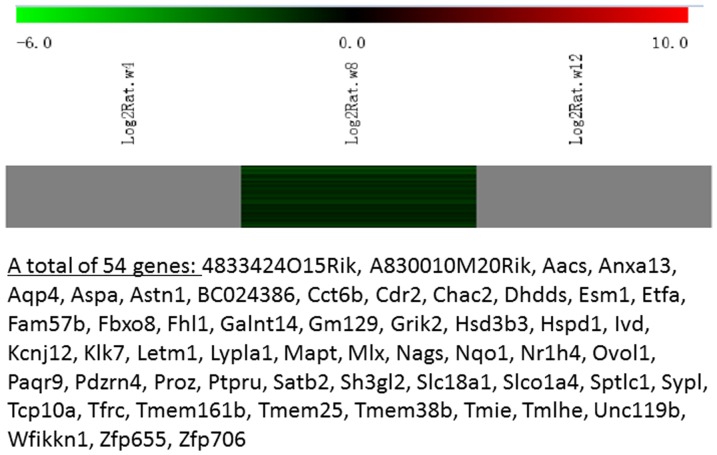
The Heat Map and the list of genes which were down-regulated transiently at the middle stage (8 weeks of age). There were a total of 54 genes in the list.

Messenger RNA was extracted from the renal cortices of three WT and three Tg26 mice at 4, 8, and 12 weeks of age for mRNA-sequencing. First, we analyzed the static levels of renal mRNA expression in Tg26 mice at three time points (4, 8, and 12 weeks). PCA was first performed to assess the sample correlations using the expression data of all the genes (Figure 1). Disease-specific changes in gene expression were determined by comparing against WT mice's gene expression to account for biopsy- and aging-related effects on gene expression. Comparisons of the gene expression profiles at three time points revealed several patterns of disease-specific changes in gene expression over the course of the disease (Figure 2). Gene ontology and pathway analyses were performed on datasets with more than 50 differentially expressed genes. We found that some genes were either consistently up-regulated or consistently down-regulated at all three time points (Figure 3 and 4). Genes that were consistently up-regulated at all three time points are involved in cytokine and Toll-like receptor pathways, immune response, inflammation, and defense mechanism (Tables S1 and S2 in File S1). Only 4 genes were consistently down-regulated at all three time points. Thirty-five genes and 1,583 genes were up-regulated only at the 4 week (Figure 5) and 12 week (Figure 6) time points, respectively. Much more genes were altered at the later stage (12 week) than early stage (4 week), possible due to secondary responses to the injury. Analyses of the dataset in Figures 6 found these genes were involved mostly in oxidative stress and RNA processing (Table S3 and S4 in File S1). In addition, we also identified a group of genes (313 genes) that were up-regulated transiently at 8 week (Figure 7). These genes are also involved in inflammation and immune defense, as well as regulation of cytoskeleton and cell adhesion (Table S5 and S6 in File S1). Twenty genes were down-regulated only at 4 week (Figure 8) and 484 genes were down-regulated only at 12 week (Figure 9). Both fatty acid and retinoid metabolisms have been shown to have renal protective effects [12] [13]. Interestingly, the 486 down-regulated genes at 12 week are involved mostly in fatty acid, retinoid, and vitamin A metabolism (Table S7 and S8 in File S1). There were a total of 54 genes which were down-regulated transiently at 8 week (Figure 10). These genes are mostly involved in cellular metabolism and immune response (Table S9 and S10 in File S1).

### Dynamic changes in renal gene expression of Tg26 mice between 4 and 8 week and 8 and 12 week

**Figure 11:**
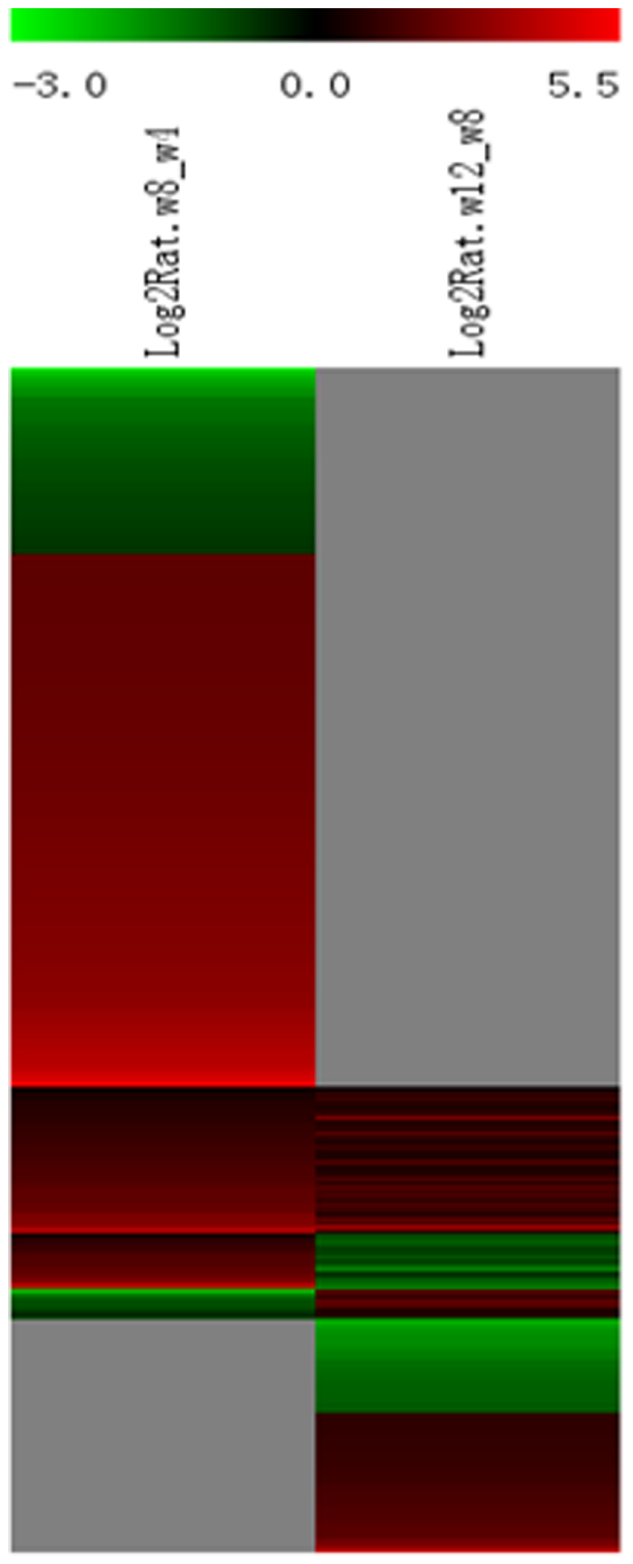
The Heat Map of the genes with dynamic changes between 4–8 weeks and 8–12 weeks of age.

Since we performed serial sampling of kidney tissue from each mouse, we were able to analyze the dynamic changes in gene expression over the course of the disease. We determined the average of changes in expression between 4 and 8 week and between 8 and 12 week for Tg26 and WT mice. This analysis identifies temporal changes in gene expression during disease progression in a dynamic manner. In addition, serial sampling of renal tissue from the same mouse permits pair-wise comparison of gene expression between different stages of the disease and avoids large inter-animal variations in gene expression. Using this analysis, we identified several patterns of changes of gene expression profiles (Figure 11). Eight four genes which were down-regulated from 4 to 8 week are involved mostly in lipid and fatty acid metabolism (Table S1 in File S2, Tables S11 and S12 in File S1). Two hundred and forty genes which were up-regulated from 4 to 8 week (Tables S2 in File S2, Tables S13 and S14 in File S1) and 66 genes which were up-regulated at both 4-to-8 week and 8-to-12 week intervals (Table S3 in File S2; Tables S15 and S16 in File S1) are all related to immune response and cytokine activation. Twenty five genes were up-regulated from 4 to 8 week but down-regulated from 8 to 12 week; and 14 genes were down-regulated from 4 to 8 week and up-regulated from 8 to 12 week (Tables S4-5 in File S2). The expression for forty two genes was not significantly different between 4 and 8 week, but was down-regulated from 8 to12 week (Table S6 in File S2). Sixty three genes were not different between 4 and 8 week, but was up-regulated from 8 to 12 weeks; and these genes are involved mostly in TGF-beta signaling and fibrosis (Tables S7 in File S2, Tables S17, and S18 in File S1).

### Validation of gene expression by real-time PCR in mouse kidneys

**Figure 12:**
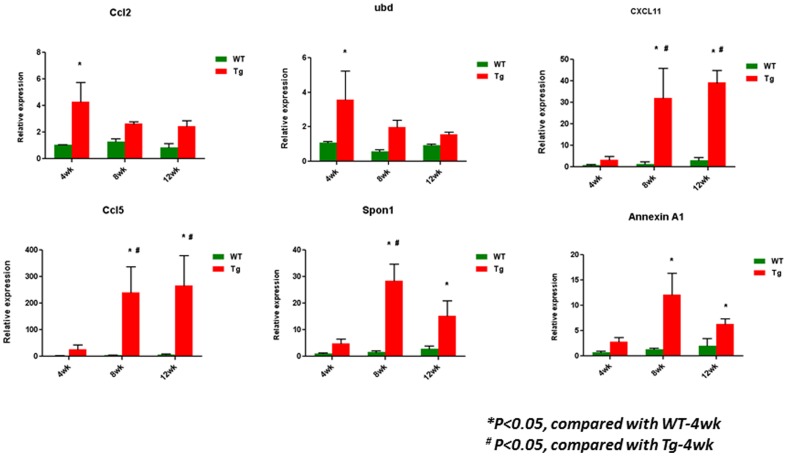
Validation of the selected genes from those up-regulated at all three time points by real-time PCR in kidney cortices of these mice. *P<0.05, compared with WT-4wk; ^#^P<0.05, compared with Tg-4wk; n = 3.

**Figure 13:**
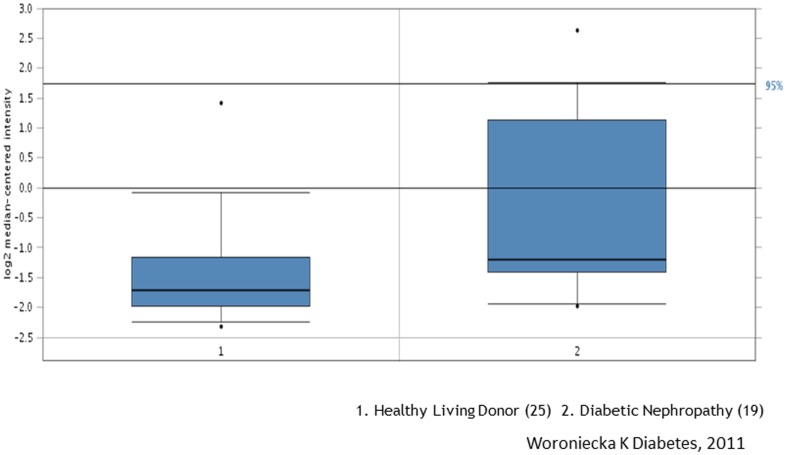
mRNA levels of Spon1 between diabetic kidneys and control kidneys. The data were obtained from Nephromine.org based on the datasets from the published studies [19].

**Figure 14:**
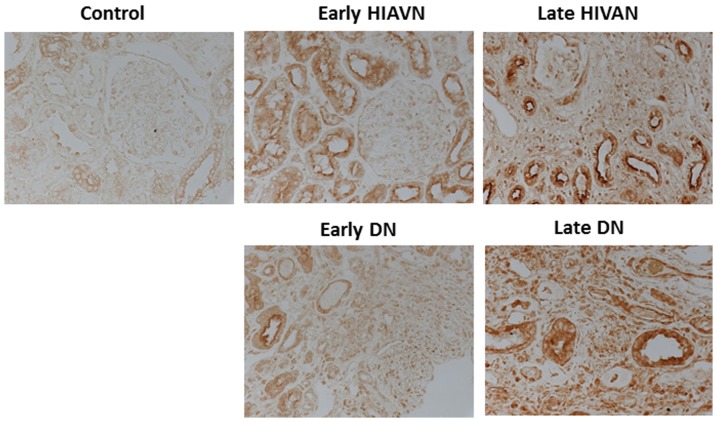
Immunostaining of Spon1 in kidney biopsies from patients with nephrectomy (control), early and late HIVAN and DN. The representative pictures from five individual patients are shown.

We selected the top 6 genes (Ccl2, Ccl5, Cxcl11, Ubd, Anxa1, and Spon1) which were consistently higher in Tg26 than WT kidney at all 3 time points for validation because we believe these genes are likely to be involved in both disease initiation and progression. We confirmed that these genes were indeed higher in the disease Tg26 kidney than control WT kidney by real-time PCR (Figure 12). Ccl2 and Ccl5 are known to be involved in the regulation of inflammation [14] [15]. Ubd, which encodes a protein called Fat10, has been shown to be a pro-apoptotic and pro-inflammatory protein in HIVAN [16]. Interestingly, Anxa1 has been reported to display anti-inflammatory effects [17]. Spon1 is a novel candidate gene for hypertension [18]. However, its role in kidney disease has never been studied. When we queried the renal expression of Spon1 in human diabetic nephropathy deposited in a web-based database called Nephromine [19], we found that the mRNA level of Spon1 was significantly higher in kidneys from individuals with diabetic nephropathy compared to non-diabetic controls (Figure 13). By immunostaining, we confirmed that the protein levels of Spon1 were also markedly higher in kidneys of patients with both early and late DN, as well as HIVAN (Figure 14). Taken together, these data suggest that Spon1 may play a role in early disease development or/and late disease progression. However, the exact function of Spon1 needs to be further validated in the future studies.

## Discussion

Mechanisms underlying CKD development and progression remain largely unknown. Treatment options that effectively prevent disease development or stop disease progression are lacking. Transcriptomics is a powerful tool for identifying potential new biomarkers and drug targets. However, the application of transcriptomics in kidney disease is limited because most renal transcriptome datasets were derived from kidney biopsies of patients with advanced kidney disease at a single point in time. Here, we evaluated the profile of gene expression changes over time in an animal model of kidney disease by serial sampling of the kidney. These data from animal models provided information that is not available for human kidney diseases, as protocol serial renal biopsies are not routinely performed. Our data suggest that findings from animal studies could help us to understand human kidney disease.

Time course experiments on gene expression are increasingly popular for exploring biological processes because temporal gene expression profiles provide an important characterization of gene function, as biological systems are both developmental and dynamic [20]. Temporal gene expression profiling could provide important information on disease progression. It is likely that the dynamic changes of gene expression could provide additional information that the static levels of gene expression could not give us. Potentially, dynamic changes of gene expression could serve as better biomarkers than static levels of gene expression. However, it is not routine clinically practice to obtain multiple kidney biopsies from the same individual during different phases of disease progression and clinical therapy. One potential alternative is to examine the dynamic changes of gene expression in peripheral blood cells or urinary cells.

Most studies examining temporal changes of gene expression are limited to in vitro cultured cells and yeasts [21] [22]. Data on the temporal pattern of gene expression in the kidney is scant. Here, we profiled the transcriptome of kidneys from Tg26 mice at three time points. These three time points reflect early, middle and late stages of kidney disease in Tg26 mice, allowing us to examine the temporal changes in gene expression. Kidney biopsy samples were also obtained from WT control mice and used to account for effects related to renal biopsy and aging-related changes in gene expression. We performed two types of analysis: static differences in gene expression between Tg26 and WT mice at different time points and dynamic changes of gene expression between different time points that are attributable to the disease. Dynamic changes in gene expression and differences in gene expression at a specific time point provide related, but non-redundant, layers of inform, which could be used to correlated with biochemical and histological parameters of the disease. We believe that this kind of comprehensive analysis could help us to better delineate disease pathogenesis and identify potential new biomarkers and targets for therapy.

Analysis of static levels of gene expression profiles at early, middle, and late points identified several patterns of changes including genes up-regulated or down-regulated at all three time points, only at early stage, transiently at the middle stage, and only at the late stage. Genes that were differentially expressed at the early stage are likely to be involved in the development of the disease. Differentially expressed genes during the late stage are likely mediators or effectors of renal injury, including fibrosis. Genes increased transiently at the middle stage may serve as molecular intermediates during disease progression. However, these causal assignments of gene function in disease development and progression are purely speculative and will need further validation in future experiments. It was immediately obvious upon inspection of the data that more genes were perturbed at the late compared to the early stage of disease; and more genes were up-regulated than down-regulated in disease. We believe, on the first pass, it is more important to focus on genes that are perturbed early in disease. Therefore, we validated some of them by real-time PCR. In particularly, we were interested in Spon1, which has never been studied in kidney disease. Spon1 is a novel candidate gene for hypertension [18] and its polymorphism is associated with severity of dementia [23]. F-spondin, coded by Spon1 gene, is a multi-domain extracellular matrix (ECM) protein and a contact-repellent molecule that directs axon outgrowth and cell migration during development [24]. F-spondin is also involved in regulation of migration and differentiation of macrophages [25]. We found that Spon1 expression is significantly higher in the kidneys of subjects with diabetic nephropathy. We further validated that Spon1 protein expression was also increased in kidney biopsies from patients with DN as well as patients with both early and late stages of HIVAN. Our studies suggest that genes identified in animal models of kidney disease could provide relevant information about human kidney disease. The role of Spon1 in kidney disease requires further studies.

In addition, analysis of dynamic in gene expression from individual mouse could help to identify genes that are associated with the progression of disease in Tg26 mice. Consistent with analysis on the static levels of gene expression, dynamic analysis revealed that the up-regulated genes are involved in immune response and down-regulated genes are involved in metabolism. Genes up-regulated at the late stage are involved in renal fibrosis. Future studies are required to determine the role of these genes in disease progression.

Several important candidate pathways and genes were identified in our studies. The function of these genes and pathways need to be further studied because they may play important role in either or both disease development and progression. In addition, genes that are differentially expression at the early stage of disease could be used as biomarkers for predicting disease progression or drug targets for early intervention. It is critical for us to understand the function of different clusters of genes during disease progression because this could help us to develop specific treatment for different stages of disease by targeting a specific subnetwork of genes or pathways.

In summary, we present here data on static levels and dynamic changes of gene expression over the disease progression in Tg26 mice. Several candidate genes and pathways were identified through our analysis. They need to be further characterized to see if they can be used as biomarkers and drug targets for prevention and treatment of kidney disease. The function and pathways associated with these differentially expressed genes and their relevance in human disease will need to be further validated in the future studies.

## Methods

### Animal studies

Derivation of a transgenic mouse line (Tg26 mice) that bears a defective HIV-1 provirus lacking gag-pol (Tg26) has been described [9]. Genotyping by tail prep and PCR were performed at 2 weeks of age as described [26]. Three Tg26 mice and three control mice generated from the same litter were used in the studies. Each mouse underwent serial renal biopsies at the age of 4 weeks, 8 weeks, and then was sacrificed at age of 12 weeks by cervical dislocation after been anesthetized with ketamine. Un-restricted food and water were provided throughout the duration of the experiment. Urine samples were collected at each time point (4, 8, and 12 wk). All animal studies were performed according to the protocols approved by Institutional Animal Care and Use Committee at the Mount Sinai School of Medicine.

### Serial Renal Biopsies

Renal biopsies were performed at 4 and 8 weeks of age. Ketamine (70 mg/kg) and xylazine (12 mg/kg) mixture was used for anesthesia. Before the surgery, the back hair was shaved and the skin was disinfected with a povidone-iodine solution. An incision of approximately 10 mm was made in the lower back and the kidney was pulled out using small forceps. A slice of the kidney was removed using a sharp surgical scissor. Strict hemostasis was performed before closing the abdominal cavity. A small piece from the lower pole of the right kidney was removed at 4 weeks of age and another small piece was removed from the upper pole of the right kidney at 8 week of age. Because the similar amount of tissues was removed from the lower or upper pole of kidney the proportions of glomeruli and tubuli in these samples were similar among individual animals. This was also confirmed by the kidney histology. On the day of sacrifice at 12 week of age, the left side of the kidney (un-biopsied sided) was collected after the perfusion with PBS. All the tissue samples were submerged in RNAlater Stabilization Reagent (Qiagen 76104) immediately after biopsies and then stored at −80°C before use.

### RNA Preparation for Sequencing

Total RNA was isolated from kidney tissues by using RNeasy mini kit (Qiagen 74104) according to the manufacturer's protocol. RNA concentrations were quantified using a Nano-drop Spectrophotometer at a wavelength of 260 nm. RNA samples were then analyzed by Bioanalyzer at a concentration of 100–200 ng/ul to verify the concentration and the purity of samples. Only the samples with RNA integrity (RIN) values of >7.0 were used for mRNA sequencing at the core facility of Mount Sinai School of Medicine.

### Bioinformatics Analysis of mRNA sequence data

The RNAseq data was analyzed by following the procedure described below. Briefly, after sequence quality filtering at a cutoff of a minimum quality score Q20 in at least 90% bases, the good quality reads were aligned to several mouse reference databases including mm9 genome, RefSeq exons, splicing junction sequences as well as contamination sequences of mouse ribosomes, mitochondria and phix genome using BWA alignment algorithm [27]. The alignments with more than 2 mismatched were discarded. After filtering out the reads that aligned to contamination sequences, the reads that are uniquely aligned to the exon and splicing-junction sites for each transcript were combined to calculate as expression level for a corresponding transcript and further normalized based on reads per kilobase per million reads(RPKM) [28] in order to compare transcription levels among samples. Gene expression value was transformed to the log 2 base scale. PCA was first performed to assess the sample correlations using the expression data of all the genes. The differentially expressed genes in biopsy in Tg26 mice compared to wild type mice were identified by the R package DEGseq [29] and ANOVA for multiple group comparisons and subjected to Gene Ontology enrichment by fisher-exact test and pathway analysis using Enrichr program [30]. The gene regulation model for disease progression was built using the statistically significant expression genes at early (4 weeks), middle (8 weeks), and late (12 weeks) stages and gene clusters with same change tendency in 3 different time points were identified and subjected to Gene Ontology enrichment and pathway analysis. The read coverage of gene functional elements was also visualized by IGV tool (Integrative Genome Viewer, http://www.broadinstitute.org/igv/) from the genome alignment file.

Also, we analyzed the dynamic changes of genes between different time points in individual mice. In brief, the changes of gene expression of individual mice between week 8 and week 4 and between week 12 and week 8 were obtained for each gene, defined as Δ_week8_week4 and Δ_week12_week8. Then, unpaired limma test [31] was used to compare the delta values between Tg26 and WT mice with a p <0.05 as significant difference. The gene lists with different patterns of changes were subjected to Gene Ontology enrichment and pathway analysis.

### Urinary albuminuria excretion

Urine albumin was quantified by ELISA using a kit from Bethyl Laboratory Inc. (Houston, TX, USA). Urine creatinine levels were measured in the same samples using QuantiChrom™ Creatinine Assay Kit (DICT-500) (BioAssay Systems) according to the manufacturer instruction. The urine albumin excretion rate was expressed as the ratio of albumin to creatinine.

### Histology

After fixation with 4% paraformaldehyde overnight, kidney tissues were embedded in paraffin by American Histolabs (Gaithersberg, MD) and 3 μm thick sections were used for staining with H&E and periodic acid-Schiff (PAS) (Sigma). Histologic changed were scored by a pathologist blind to mouse genotype. The percentage of glomerulosclerosis was obtained by identifying the total number of glomeruli with any sclerosis and dividing this number by the total number of glomeruli seen. The percentage of tubular atrophy/fibrosis score was obtained by the number of tubules with either tubular atrophy or microcystic dilatation divided by the total number of tubular cross sections in a representative area or the percentage of area with fibrosis divided by the total area examined in a cross section.

### Real-time PCR

First strand cDNA was prepared from total RNA (1.5 ug) using the SuperScript™ III First-Strand Synthesis Kit (Invitrogen) and cDNA (1 μl) was amplified in triplicate using SYBR GreenER qPCR Supermix on an ABI PRISM 7900 HT (Applied Biosystems, Foster City, CA, USA). Primers were designed using PrimerBlast (http://www.ncbi.nlm.nih.gov/tools/primer-blast/) and purchased through Sigma (Table 1). The sequences of the primers are listed in the Table 2. Light cycler analysis software was used to determine crossing points using the second derivative method. Efficiency was calculated for all the designed primers using relative standard curve method. Data was normalized to housekeeping gene (GAPDH) and presented as fold increase compared to RNA isolated from WT animals using the Pfaffl method [32].

**Table 2:** Real Time PCR Primers.

Gene	Forward	Reverse
Ccl2	TTAAAAACCTGGATCGGAACCAA	GCATTAGCTTCAGATTTACGGGT
Ccl5	GCTGCTTTGCCTACCTCTCC	TCGAGTGACAAACACGACTGC
Ccl11	AGATGCACCCTGAAAGCC	AGCCAAGTCCTTGGGCGAC
Ubd	ACAGACATGGCTTCTGTCCG	TTTCGATGGGGCTTGAGGA
Anxa 1	TGTTGCTGCCTTGCACAAAGC	AGTACGCGGCCTTGATCTG
Spon 1	AGGGCTCCCTGACCAAGAAG	TACTTGGCAGTTCCACAGGC

### Immunohistochemistry

Archival Human biopsy specimens of healthy donor nephrectomies, HIVAN, and DN for IHC were collected at Mount Sinai School of Medicine under a protocol approved by its Institutional Review Board. Specimens were initially baked for 20 minutes in 55–60°C oven and then processed as previously described below. Briefly formalin-fixed and paraffin embedded sections were deparaffinized, and endogenous peroxidase was inactivated with H_2_O_2_. Sections were then blocked in 2% goat serum in phosphate buffered saline (PBS) for 1 hour at room temperature and then incubated with a rabbit anti-Spon1 antibody (Abcam) at 4°C overnight. The next day, sections were washed three times with PBS and then incubated with secondary antibody for 30 minutes. Positive staining was revealed by peroxidase-labeled streptavidin and diaminobenzidine substrate.

### Statistical analysis

Data were expressed as mean±standard deviation (*X*±SEM). The unpaired t-test was used to analyze data between two groups. The ANOVA followed by Bonferroni correction was used when more than two groups were present. All experiments were repeated at least three times, and representative experiments are shown. Statistical significance will be considered when *P*<0.05.

## Supporting Information

File S1 **Pathway analysis of differentially regulated genes.** **Table S1**: The 69 genes, which were up-regulated at all three time points, were analyzed by using Enrichr KEGG program. **Table S2**: The 69 genes, which were up-regulated at all three time points, were further analyzed by using Enrichr GO Biological Process program. **Table S3**: The 1583 genes, which were up-regulated at late stage (12 weeks), were analyzed further by using Enrichr KEGG program. **Table S4**: The 1583 genes, which were up-regulated at late stage (12 weeks), were analyzed further by using Enrichr GO Biological Process. **Table S5**: The 313 genes, which were up-regulated transiently at the middle stage (8 weeks), were analyzed further by using Enrichr KEGG program. **Table S6**: The 313 genes, which were up-regulated transiently at the middle stage (8 weeks), were analyzed further by using Enrichr GO Biological Process program. **Table S7**: The 486 genes, which were down-regulated at the late stage (12 weeks), were analyzed further by using Enrichr KEGG program. **Table S8**: The 486 genes, which were down-regulated at the late stage (12 weeks), were analyzed further by using Enrichr GO Biological Process. **Table S9**: The 54 genes, which were down-regulated transiently at 8 weeks of age, were analyzed further by using Enrichr KEGG program. **Table S10**: The 54 genes, which were down-regulated transiently at 8 weeks of age, were analyzed further by using Enrichr GO Biological Process. **Table S11**: 84 genes which were down-regulated between 4–8 weeks but no changes between 8–12 weeks further analyzed for Enrichr KEGG. **Table S12**: 84 genes which were down-regulated between 4–8 weeks but no changes between 8–12 weeks further analyzed for Enrichr Go Biological Process. **Table S13**: 240 genes which were up-regulated between 4–8 weeks but no changes between 8–12 weeks further analyzed for Enrichr KEGG. **Table S14**: 240 genes which were up-regulated between 4–8 weeks but no changes between 8–12 weeks further analyzed by GO Biological Process. **Table S15**: 66 genes which were up-regulated between 4–8 weeks and between 8–12 weeks further analyzed for Enrichr KEGG. **Table S16**: 66 genes which were up-regulated between 4–8 weeks and between 8–12 weeks further analyzed by GO Biological Process. **Table S17**: 63 genes which were no changes between 4–8 weeks but up-regulated between 8–12 weeks further analyzed for Enrichr KEGG. **Table S18**: 63 genes which were no changes between 4–8 weeks but up-regulated between 8–12 weeks further analyzed for Enrichr GO Biological Process.
(PPTX)

File S2 **Lists of genes differentially regulated between different time points (dynamic changes).** **Table S1**: Genes down-regulated from 4 to 8 weeks but no changes from 8 weeks to 12 weeks. **Table S2**: Genes up-regulated from 4 to 8 weeks but no changes from 8 weeks to 12 weeks. **Table S3**: Genes up-regulated from 4 to 8 weeks and from 8 weeks to 12 weeks. **Table S4**: Genes up-regulated from 4 to 8 weeks but down-regulated from 8 weeks to 12 weeks. **Table S5**: Genes down-regulated from 4 to 8 weeks but up-regulated from 8 weeks to 12 weeks. **Table S6**: Genes no changes from 4 to 8 weeks but down-regulated from 8 weeks to 12 weeks. **Table S7**: Genes no changes from 4 to 8 weeks but up-regulated from 8 weeks to 12 weeks.
(DOCX)

## References

[1] Appel LJ, Wright JT Jr, Greene T, Kusek JW, Lewis JB, et al (2008) Long-term effects of renin-angiotensin system-blocking therapy and a low blood pressure goal on progression of hypertensive chronic kidney disease in African Americans. Arch Intern Med 168: 832–839. 18443258 10.1001/archinte.168.8.832 PMC3870204

[2] Sharma P, Blackburn RC, Parke CL, McCullough K, Marks A, et al. (2011) Angiotensin-converting enzyme inhibitors and angiotensin receptor blockers for adults with early (stage 1 to 3) non-diabetic chronic kidney disease. Cochrane Database Syst Rev: CD007751. 10.1002/14651858.CD007751.pub2 21975774

[3] Martini S, Eichinger F, Nair V, Kretzler M (2008) Defining human diabetic nephropathy on the molecular level: integration of transcriptomic profiles with biological knowledge. Rev Endocr Metab Disord 9: 267–274. 18704688 10.1007/s11154-008-9103-3 PMC2597685

[4] Berthier CC, Zhang H, Schin M, Henger A, Nelson RG, et al (2009) Enhanced expression of Janus kinase-signal transducer and activator of transcription pathway members in human diabetic nephropathy. Diabetes 58: 469–477. 19017763 10.2337/db08-1328 PMC2628622

[5] Hodgin JB, Borczuk AC, Nasr SH, Markowitz GS, Nair V, et al (2010) A molecular profile of focal segmental glomerulosclerosis from formalin-fixed, paraffin-embedded tissue. Am J Pathol 177: 1674–1686. 20847290 10.2353/ajpath.2010.090746 PMC2947265

[6] Ju W, Eichinger F, Bitzer M, Oh J, McWeeney S, et al (2009) Renal gene and protein expression signatures for prediction of kidney disease progression. Am J Pathol 174: 2073–2085. 19465643 10.2353/ajpath.2009.080888 PMC2684173

[7] Bhavnani SK, Eichinger F, Martini S, Saxman P, Jagadish HV, et al (2009) Network analysis of genes regulated in renal diseases: implications for a molecular-based classification. BMC Bioinformatics 10 Suppl 9: S3. 10.1186/1471-2105-10-S9-S3 PMC2745690 19761573

[8] Brosius FC 3rd, Alpers CE, Bottinger EP, Breyer MD, Coffman TM, et al (2009) Mouse models of diabetic nephropathy. J Am Soc Nephrol 20: 2503–2512. 19729434 10.1681/ASN.2009070721 PMC4075053

[9] Dickie P, Felser J, Eckhaus M, Bryant J, Silver J, et al (1991) HIV-associated nephropathy in transgenic mice expressing HIV-1 genes. Virology 185: 109–119. 1926769 10.1016/0042-6822(91)90759-5

[10] Lu TC, He JC, Klotman P (2006) Animal models of HIV-associated nephropathy. Curr Opin Nephrol Hypertens 15: 233–237. 16609288 10.1097/01.mnh.0000222688.69217.8e

[11] Kopp JB, Klotman ME, Adler SH, Bruggeman LA, Dickie P, et al (1992) Progressive glomerulosclerosis and enhanced renal accumulation of basement membrane components in mice transgenic for human immunodeficiency virus type 1 genes. Proc Natl Acad Sci U S A 89: 1577–1581. 1542649 10.1073/pnas.89.5.1577 PMC48495

[12] Ratnam KK, Feng X, Chuang PY, Verma V, Lu TC, et al Role of the retinoic acid receptor-alpha in HIV-associated nephropathy. Kidney Int 79: 624–634. 21150871 10.1038/ki.2010.470 PMC3050085

[13] Wang H, Liu H, Jia Z, Olsen C, Litwin S, et al (2010) Nitro-oleic acid protects against endotoxin-induced endotoxemia and multiorgan injury in mice. Am J Physiol Renal Physiol 298: F754–762. 20032118 10.1152/ajprenal.00439.2009 PMC2838591

[14] Tesch GH (2008) MCP-1/CCL2: a new diagnostic marker and therapeutic target for progressive renal injury in diabetic nephropathy. Am J Physiol Renal Physiol 294: F697–701. 18272603 10.1152/ajprenal.00016.2008

[15] Krensky AM, Ahn YT (2007) Mechanisms of disease: regulation of RANTES (CCL5) in renal disease. Nat Clin Pract Nephrol 3: 164–170. 17322928 10.1038/ncpneph0418 PMC2702760

[16] Gong P, Canaan A, Wang B, Leventhal J, Snyder A, et al (2009) The ubiquitin-like protein FAT10 mediates NF-kappaB activation. J Am Soc Nephrol 21: 316–326. 19959714 10.1681/ASN.2009050479 PMC2834541

[17] Araujo LP, Truzzi RR, Mendes GE, Luz MA, Burdmann EA, et al (2010) Interaction of the anti-inflammatory annexin A1 protein and tacrolimus immunosuppressant in the renal function of rats. Am J Nephrol 31: 527–533. 20484890 10.1159/000309756

[18] Clemitson JR, Dixon RJ, Haines S, Bingham AJ, Patel BR, et al (2007) Genetic dissection of a blood pressure quantitative trait locus on rat chromosome 1 and gene expression analysis identifies SPON1 as a novel candidate hypertension gene. Circ Res 100: 992–999. 17332427 10.1161/01.RES.0000261961.41889.9c PMC3533402

[19] Woroniecka KI, Park AS, Mohtat D, Thomas DB, Pullman JM, et al (2011) Transcriptome analysis of human diabetic kidney disease. Diabetes 60: 2354–2369. 21752957 10.2337/db10-1181 PMC3161334

[20] Bar-Joseph Z (2004) Analyzing time series gene expression data. Bioinformatics 20: 2493–2503. 15130923 10.1093/bioinformatics/bth283

[21] Sharif O, Bolshakov VN, Raines S, Newham P, Perkins ND (2007) Transcriptional profiling of the LPS induced NF-kappaB response in macrophages. BMC Immunol 8: 1. 17222336 10.1186/1471-2172-8-1 PMC1781469

[22] Spellman PT, Sherlock G, Zhang MQ, Iyer VR, Anders K, et al (1998) Comprehensive identification of cell cycle-regulated genes of the yeast Saccharomyces cerevisiae by microarray hybridization. Mol Biol Cell 9: 3273–3297. 9843569 10.1091/mbc.9.12.3273 PMC25624

[23] Jahanshad N, Rajagopalan P, Hua X, Hibar DP, Nir TM, et al (2013) Genome-wide scan of healthy human connectome discovers SPON1 gene variant influencing dementia severity. Proc Natl Acad Sci U S A 110: 4768–4773. 23471985 10.1073/pnas.1216206110 PMC3606977

[24] Tan K, Lawler J (2011) The structure of the Ca(2)+-binding, glycosylated F-spondin domain of F-spondin - A C2-domain variant in an extracellular matrix protein. BMC Struct Biol 11: 22. 21569239 10.1186/1472-6807-11-22 PMC3117680

[25] Oka H, Mori M, Kihara H (2011) F-spondin inhibits migration and differentiation of osteoclastic precursors. J Periodontol 82: 1776–1783. 21488757 10.1902/jop.2011.110111

[26] Feng X, Lu TC, Chuang PY, Fang W, Ratnam K, et al (2009) Reduction of Stat3 Activity Attenuates HIV-Induced Kidney Injury. J Am Soc Nephrol. 10.1681/ASN.2008080879 PMC2754106 19608706

[27] Li H, Durbin R (2009) Fast and accurate short read alignment with Burrows-Wheeler transform. Bioinformatics 25: 1754–1760. 19451168 10.1093/bioinformatics/btp324 PMC2705234

[28] Mortazavi A, Williams BA, McCue K, Schaeffer L, Wold B (2008) Mapping and quantifying mammalian transcriptomes by RNA-Seq. Nat Methods 5: 621–628. 18516045 10.1038/nmeth.1226 PMC13303166

[29] Wang L, Feng Z, Wang X, Zhang X (2010) DEGseq: an R package for identifying differentially expressed genes from RNA-seq data. Bioinformatics 26: 136–138. 19855105 10.1093/bioinformatics/btp612

[30] Chen EY, Tan CM, Kou Y, Duan Q, Wang Z, et al (2013) Enrichr: interactive and collaborative HTML5 gene list enrichment analysis tool. BMC Bioinformatics 14: 128. 23586463 10.1186/1471-2105-14-128 PMC3637064

[31] Berger DK, Crampton BG, Hein I, Vos W (2007) Screening of cDNA libraries on glass slide microarrays. Methods Mol Biol 382: 177–203. 18220232 10.1007/978-1-59745-304-2_12

[32] Pfaffl MW (2001) A new mathematical model for relative quantification in real-time RT-PCR. Nucleic Acids Res 29: e45. 11328886 10.1093/nar/29.9.e45 PMC55695

