# Supplementary material for: Temporal Profile of the Renal Transcriptome of HIV-1 Transgenic Mice during Disease Progression
Source: PLoS One. 2014 Mar 25;9(3):e93019. doi: 10.1371/journal.pone.0093019 (PMC3965528; doi:10.1371/journal.pone.0093019)
Supplement: File S2 — Lists of genes differentially regulated between different time points (dynamic changes). Table S1: Genes down-regulated from 4 to 8 weeks but no changes from 8 weeks to 12 weeks. Table S2: Genes up-regulated from 4 to 8 weeks but no changes from 8 weeks to 12 weeks. Table S3: Genes up-regulated from 4 to 8 weeks and from 8 weeks to 12 weeks. Table S4: Genes up-regulated from 4 to 8 weeks but down-regulated from 8 weeks to 12 weeks. Table S5: Genes down-regulated from 4 to 8 weeks but up-regulated from 8 weeks to 12 weeks. Table S6: Genes no changes from 4 to 8 weeks but down-regulated from 8 weeks to 12 weeks. Table S7: Genes no changes from 4 to 8 weeks but up-regulated from 8 weeks to 12 weeks. (DOCX) [file pone.0093019.s002.docx]

**Table S1: Genes down-regulated from 4 to 8 weeks but no changes from 8 weeks to 12 weeks**

| Symbol | RefSeq | Gene Name | pvalue.w8_w4 | Log2Rat.w8_w4 |
| --- | --- | --- | --- | --- |
| BC014805 | NM_146232 | cDNA sequence BC014805 | 0.042988 | -2.99592 |
| Upp2 | NM_029692 | uridine phosphorylase 2 | 0.017421 | -2.42754 |
| C4a | NM_011413 | complement component 4A (Rodgers blood group) | 0.024093 | -2.28505 |
| Cndp1 | NM_177450 | carnosine dipeptidase 1 (metallopeptidase M20 family) | 0.038669 | -2.27193 |
| Mchr1 | NM_145132 | melanin-concentrating hormone receptor 1 | 0.019982 | -2.03985 |
| Angptl7 | NM_001039554 | angiopoietin-like 7 | 0.027717 | -2.01809 |
| Tmprss11c | NM_001030297 | transmembrane protease, serine 11c | 0.002304 | -1.93773 |
| Tc2n | NM_028924 | tandem C2 domains, nuclear | 0.020618 | -1.90141 |
| Aqp4 | NM_009700 | aquaporin 4 | 0.006847 | -1.80615 |
| Mfsd2a | NM_029662 | major facilitator superfamily domain containing 2A | 0.02835 | -1.73716 |
| Srd5a2 | NM_053188 | steroid 5 alpha-reductase 2 | 0.042691 | -1.66912 |
| Agxt2l1 | NM_027907 | alanine-glyoxylate aminotransferase 2-like 1 | 0.047325 | -1.66661 |
| 4930502E18Rik | NM_029142 | RIKEN cDNA 4930502E18 gene | 0.007381 | -1.62761 |
| Esm1 | NM_023612 | endothelial cell-specific molecule 1 | 0.004847 | -1.45998 |
| Acot12 | NM_028790 | acyl-CoA thioesterase 12 | 0.047535 | -1.37187 |
| Nr1h4 | NM_009108 | nuclear receptor subfamily 1, group H, member 4 | 0.037202 | -1.36999 |
| Nrg4 | NM_032002 | neuregulin 4 | 0.033752 | -1.36838 |
| Syn3 | NM_013722 | synapsin III | 0.017208 | -1.33979 |
| Mpped1 | NM_172610 | metallophosphoesterase domain containing 1 | 0.013351 | -1.31482 |
| Mmp10 | NM_019471 | matrix metallopeptidase 10 | 0.001083 | -1.29662 |
| C030002C11Rik | NR_015566 | RIKEN cDNA C030002C11 gene | 0.005617 | -1.27094 |
| Cpeb3 | NM_198300 | cytoplasmic polyadenylation element binding protein 3 | 0.029655 | -1.27091 |
| A330035P11Rik | NR_015586 | RIKEN cDNA A330035P11 gene | 0.036809 | -1.26197 |
| Tet2 | NM_001040400 | tet oncogene family member 2 | 0.029247 | -1.2553 |
| Klk7 | NM_011872 | kallikrein related-peptidase 7 (chymotryptic, stratum corneum) | 0.041185 | -1.23185 |
| Nudt19 | NM_033080 | nudix (nucleoside diphosphate linked moiety X)-type motif 19 | 0.013011 | -1.20485 |
| A330040F15Rik | NR_015503 | RIKEN cDNA A330040F15 gene | 0.011718 | -1.14509 |
| Gm13154 | NM_001014397 | predicted gene 13154 | 0.026823 | -1.12246 |
| Tmem207 | NM_001101640 | transmembrane protein 207 | 0.034089 | -1.12071 |
| Hsd3b2 | NM_153193 | hydroxy-delta-5-steroid dehydrogenase, 3 beta- and steroid delta-isomerase 2 | 0.029875 | -1.11109 |
| Rnf24 | NM_178607 | ring finger protein 24 | 0.032436 | -1.0842 |
| Afmid | NM_027827 | arylformamidase | 0.037511 | -1.06534 |
| Katnal1 | NM_153572 | katanin p60 subunit A-like 1 | 0.027955 | -1.06294 |
| Kcnk5 | NM_021542 | potassium channel, subfamily K, member 5 | 0.015405 | -1.05892 |
| Aqp1 | NM_007472 | aquaporin 1 | 0.019091 | -1.0579 |
| Fcamr | NM_144960 | Fc receptor, IgA, IgM, high affinity | 0.049943 | -1.03768 |
| Ghr | NM_010284 | growth hormone receptor | 0.039885 | -1.03149 |
| B3galt2 | NM_020025 | UDP-Gal:betaGlcNAc beta 1,3-galactosyltransferase, polypeptide 2 | 0.036466 | -1.01616 |
| Coq10b | NM_026424 | coenzyme Q10 homolog B (S. cerevisiae) | 0.047777 | -1.00426 |
| Sucnr1 | NM_032400 | succinate receptor 1 | 0.008412 | -0.99673 |
| Ampd1 | NM_001033303 | adenosine monophosphate deaminase 1 (isoform M) | 0.042323 | -0.96901 |
| Wfikkn1 | NM_001100454 | WAP, FS, Ig, KU, and NTR-containing protein 1 | 0.033569 | -0.93472 |
| Prok1 | NM_001044382 | prokineticin 1 | 0.022848 | -0.92335 |
| Nol4 | NM_199024 | nucleolar protein 4 | 0.04071 | -0.92252 |
| Nr2e3 | NM_013708 | nuclear receptor subfamily 2, group E, member 3 | 0.013233 | -0.91557 |
| Lypla1 | NM_008866 | lysophospholipase 1 | 0.041754 | -0.90266 |
| Galnt11 | NM_144908 | UDP-N-acetyl-alpha-D-galactosamine:polypeptide N-acetylgalactosaminyltransferase 11 | 0.043919 | -0.8978 |
| Rgl1 | NM_016846 | ral guanine nucleotide dissociation stimulator,-like 1 | 0.030589 | -0.89777 |
| Glyctk | NM_174846 | glycerate kinase | 0.024324 | -0.8951 |
| Nags | NM_178053 | N-acetylglutamate synthase | 0.013756 | -0.8921 |
| Fmo1 | NM_010231 | flavin containing monooxygenase 1 | 0.032557 | -0.87965 |
| Dbt | NM_010022 | dihydrolipoamide branched chain transacylase E2 | 0.049877 | -0.86282 |
| Rn4.5s | NR_002841 | 4.5S RNA | 0.033154 | -0.8293 |
| Gas2 | NM_008087 | growth arrest specific 2 | 0.019132 | -0.82862 |
| Dgkb | NM_178681 | diacylglycerol kinase, beta | 0.043459 | -0.82748 |
| A630095E13Rik | NM_001033325 | RIKEN cDNA A630095E13 gene | 0.017116 | -0.80787 |
| Rab3b | NM_023537 | RAB3B, member RAS oncogene family | 0.004906 | -0.80723 |
| Chpt1 | NR_027477 | choline phosphotransferase 1 | 0.045827 | -0.77873 |
| Pdzrn4 | NM_001164594 | PDZ domain containing RING finger 4 | 0.045387 | -0.76988 |
| Tmie | NM_146260 | transmembrane inner ear | 0.048369 | -0.76771 |
| Gm7120 | NM_001039244 | predicted gene 7120 | 0.035474 | -0.76484 |
| Kcnc1 | NM_008421 | potassium voltage gated channel, Shaw-related subfamily, member 1 | 0.04748 | -0.76427 |
| Foxq1 | NM_008239 | forkhead box Q1 | 0.036475 | -0.75769 |
| Acsm2 | NM_146197 | acyl-CoA synthetase medium-chain family member 2 | 0.038712 | -0.75666 |
| Tnnt1 | NM_011618 | troponin T1, skeletal, slow | 0.033304 | -0.75578 |
| Anxa13 | NM_027211 | annexin A13 | 0.01999 | -0.75522 |
| Hspb7 | NM_013868 | heat shock protein family, member 7 (cardiovascular) | 0.008776 | -0.75247 |
| Vmn2r102 | NM_001104564 | vomeronasal 2, receptor 102 | 0.016425 | -0.75185 |
| Ovol1 | NM_019935 | OVO homolog-like 1 (Drosophila) | 0.024592 | -0.7379 |
| Efcab2 | NM_026626 | EF-hand calcium binding domain 2 | 0.049186 | -0.7122 |
| Cml1 | NM_023160 | camello-like 1 | 0.022384 | -0.70226 |
| Lactb2 | NM_145381 | lactamase, beta 2 | 0.046317 | -0.69949 |
| Mcf2 | NM_133197 | mcf.2 transforming sequence | 0.02414 | -0.69719 |
| Nat2 | NM_010874 | N-acetyltransferase 2 (arylamine N-acetyltransferase) | 0.024653 | -0.69392 |
| Gzmc | NM_010371 | granzyme C | 0.026635 | -0.68855 |
| Bphl | NM_026512 | biphenyl hydrolase-like (serine hydrolase, breast epithelial mucin-associated antigen) | 0.043742 | -0.6738 |
| Tmigd1 | NM_025655 | transmembrane and immunoglobulin domain containing 1 | 0.033873 | -0.64673 |
| Lrriq1 | NM_029134 | leucine-rich repeats and IQ motif containing 1 | 0.02593 | -0.6405 |
| Snx29 | NM_028964 | sorting nexin 29 | 0.045112 | -0.63839 |
| Gm13285 | NM_001161609 | predicted gene 13285 | 0.046515 | -0.63267 |
| Chac2 | NM_026527 | ChaC, cation transport regulator homolog 2 (E. coli) | 0.014309 | -0.60887 |
| P2ry1 | NM_008772 | purinergic receptor P2Y, G-protein coupled 1 | 0.014762 | -0.60077 |
| Gm5538 | NM_001101531 | predicted gene 5538 | 0.047097 | -0.59447 |
| 4930434E21Rik | NM_029440 | RIKEN cDNA 4930434E21 gene | 0.04734 | -0.58963 |

**Table S2: Genes up-regulated from 4 to 8 weeks but no changes from 8 weeks to 12 weeks**

| Symbol | RefSeq | Gene Name | pvalue.w8_w4 | Log2Rat.w8_w4 |
| --- | --- | --- | --- | --- |
| Thy1 | NM_009382 | thymus cell antigen 1, theta | 0.003748 | 2.004878 |
| Ptpn7 | NM_177081 | protein tyrosine phosphatase, non-receptor type 7 | 0.000415 | 2.010108 |
| Adamdec1 | NM_021475 | ADAM-like, decysin 1 | 0.023211 | 2.016316 |
| Serpinh1 | NM_009825 | serine (or cysteine) peptidase inhibitor, clade H, member 1 | 0.046929 | 2.019269 |
| Cd14 | NM_009841 | CD14 antigen | 0.005311 | 2.024271 |
| Psmb8 | NM_010724 | proteasome (prosome, macropain) subunit, beta type 8 (large multifunctional peptidase 7) | 0.000815 | 2.027782 |
| Hebp2 | NM_019487 | heme binding protein 2 | 0.005265 | 2.028199 |
| Cd74 | NM_010545 | CD74 antigen (invariant polypeptide of major histocompatibility complex, class II antigen-associated) | 5.53E-05 | 2.03307 |
| BC005764 | NM_181681 | cDNA sequence BC005764 | 0.000679 | 2.033726 |
| Pglyrp2 | NM_021319 | peptidoglycan recognition protein 2 | 0.023972 | 2.036137 |
| Cenpf | NM_001081363 | centromere protein F | 0.028604 | 2.037817 |
| Crip1 | NM_007763 | cysteine-rich protein 1 (intestinal) | 0.005867 | 2.038711 |
| H2-Eb1 | NM_010382 | histocompatibility 2, class II antigen E beta | 0.001857 | 2.040199 |
| Crlf1 | NM_018827 | cytokine receptor-like factor 1 | 0.001002 | 2.049295 |
| Clec10a | NM_010796 | C-type lectin domain family 10, member A | 0.001512 | 2.051365 |
| Map4k1 | NM_008279 | mitogen-activated protein kinase kinase kinase kinase 1 | 0.003899 | 2.051725 |
| Clec4a1 | NM_199311 | C-type lectin domain family 4, member a1 | 0.002384 | 2.051873 |
| Nfam1 | NM_028728 | Nfat activating molecule with ITAM motif 1 | 0.00031 | 2.053391 |
| Hpgds | NM_019455 | hematopoietic prostaglandin D synthase | 0.039758 | 2.053867 |
| H2-Aa | NM_010378 | histocompatibility 2, class II antigen A, alpha | 7.74E-05 | 2.054918 |
| Nfil3 | NM_017373 | nuclear factor, interleukin 3, regulated | 0.004994 | 2.063371 |
| Nfe2 | NM_008685 | nuclear factor, erythroid derived 2 | 0.041376 | 2.066342 |
| Mfap2 | NM_008546 | microfibrillar-associated protein 2 | 0.007405 | 2.069023 |
| Rgs16 | NM_011267 | regulator of G-protein signaling 16 | 0.008466 | 2.070891 |
| H1fx | NM_198622 | H1 histone family, member X | 0.035102 | 2.072552 |
| Col1a2 | NM_007743 | collagen, type I, alpha 2 | 0.001653 | 2.076548 |
| Pyhin1 | NM_175026 | pyrin and HIN domain family, member 1 | 0.00032 | 2.0769 |
| Tcf7 | NM_009331 | transcription factor 7, T-cell specific | 0.000733 | 2.084522 |
| Tnfaip8l2 | NM_027206 | tumor necrosis factor, alpha-induced protein 8-like 2 | 0.000295 | 2.085974 |
| Hcls1 | NM_008225 | hematopoietic cell specific Lyn substrate 1 | 0.000579 | 2.086127 |
| 4632428N05Rik | NM_028732 | RIKEN cDNA 4632428N05 gene | 0.000257 | 2.086338 |
| Col6a2 | NM_146007 | collagen, type VI, alpha 2 | 0.025526 | 2.091327 |
| Lrmp | NM_008511 | lymphoid-restricted membrane protein | 0.001213 | 2.092821 |
| C1qb | NM_009777 | complement component 1, q subcomponent, beta polypeptide | 6.62E-05 | 2.096166 |
| Fam105a | NM_198301 | family with sequence similarity 105, member A | 0.000382 | 2.097178 |
| Rnase6 | NM_030098 | ribonuclease, RNase A family, 6 | 0.028908 | 2.097429 |
| Ercc6l | NM_146235 | excision repair cross-complementing rodent repair deficiency complementation group 6 - like | 0.004764 | 2.097557 |
| Ctsk | NM_007802 | cathepsin K | 0.001542 | 2.100672 |
| Tcf19 | NM_025674 | transcription factor 19 | 0.01255 | 2.102367 |
| 2310014H01Rik | NM_175242 | RIKEN cDNA 2310014H01 gene | 0.001035 | 2.105221 |
| Lrrc25 | NM_153074 | leucine rich repeat containing 25 | 0.000165 | 2.106267 |
| Emilin1 | NM_133918 | elastin microfibril interfacer 1 | 0.001464 | 2.109276 |
| Ccl5 | NM_013653 | chemokine (C-C motif) ligand 5 | 0.030273 | 2.109669 |
| Parvg | NM_022321 | parvin, gamma | 0.000106 | 2.109843 |
| Aldh1b1 | NM_028270 | aldehyde dehydrogenase 1 family, member B1 | 0.007324 | 2.111029 |
| Htra3 | NM_030127 | HtrA serine peptidase 3 | 0.020326 | 2.11757 |
| Hpse | NM_152803 | heparanase | 0.001845 | 2.122223 |
| Lat | NM_010689 | linker for activation of T cells | 0.0045 | 2.123884 |
| Itgb3 | NM_016780 | integrin beta 3 | 0.001754 | 2.127243 |
| Tnfrsf4 | NM_011659 | tumor necrosis factor receptor superfamily, member 4 | 0.017738 | 2.129465 |
| Aif1 | NM_019467 | allograft inflammatory factor 1 | 0.002558 | 2.133644 |
| Klk8 | NM_008940 | kallikrein related-peptidase 8 | 0.003334 | 2.133955 |
| Pstpip1 | NM_011193 | proline-serine-threonine phosphatase-interacting protein 1 | 0.039302 | 2.137834 |
| C3ar1 | NM_009779 | complement component 3a receptor 1 | 0.013096 | 2.138693 |
| Card9 | NM_001037747 | caspase recruitment domain family, member 9 | 0.024591 | 2.141219 |
| Lilrb3 | NM_011095 | leukocyte immunoglobulin-like receptor, subfamily B (with TM and ITIM domains), member 3 | 7.01E-05 | 2.141832 |
| Cd37 | NM_007645 | CD37 antigen | 0.000551 | 2.145406 |
| Cd163 | NM_053094 | CD163 antigen | 0.020812 | 2.147473 |
| Cd22 | NM_009845 | CD22 antigen | 0.003274 | 2.153435 |
| Coro1a | NM_009898 | coronin, actin binding protein 1A | 0.001517 | 2.154491 |
| Cxcr6 | NM_030712 | chemokine (C-X-C motif) receptor 6 | 0.001083 | 2.156923 |
| Hn1 | NM_008258 | hematological and neurological expressed sequence 1 | 0.026641 | 2.158377 |
| Lsp1 | NM_019391 | lymphocyte specific 1 | 0.00032 | 2.160184 |
| Smoc2 | NM_022315 | SPARC related modular calcium binding 2 | 0.043483 | 2.174886 |
| Gm11428 | NM_001081957 | predicted gene 11428 | 0.00094 | 2.176808 |
| Clec7a | NM_020008 | C-type lectin domain family 7, member a | 0.000705 | 2.179767 |
| C1qa | NM_007572 | complement component 1, q subcomponent, alpha polypeptide | 0.002866 | 2.181737 |
| Ptprcap | NM_016933 | protein tyrosine phosphatase, receptor type, C polypeptide-associated protein | 0.000228 | 2.189199 |
| Espl1 | NM_001014976 | extra spindle poles-like 1 (S. cerevisiae) | 0.007956 | 2.192726 |
| Mcm5 | NM_008566 | minichromosome maintenance deficient 5, cell division cycle 46 (S. cerevisiae) | 0.01024 | 2.195003 |
| Fbln1 | NM_010180 | fibulin 1 | 0.000703 | 2.203513 |
| Rinl | NM_177158 | Ras and Rab interactor-like | 0.010512 | 2.208391 |
| Lgals3 | NM_010705 | lectin, galactose binding, soluble 3 | 0.009921 | 2.21429 |
| Il2ra | NM_008367 | interleukin 2 receptor, alpha chain | 0.00181 | 2.215048 |
| H2-Ab1 | NM_207105 | histocompatibility 2, class II antigen A, beta 1 | 0.0005 | 2.217184 |
| Folr4 | NM_176807 | folate receptor 4 (delta) | 0.011895 | 2.230408 |
| Hba-a1 | NM_008218 | hemoglobin alpha, adult chain 1 | 0.005879 | 2.231575 |
| Nrm | NM_134122 | nurim (nuclear envelope membrane protein) | 0.00993 | 2.232096 |
| Ccnb2 | NM_007630 | cyclin B2 | 0.020852 | 2.233021 |
| Gpr68 | NM_175493 | G protein-coupled receptor 68 | 0.000849 | 2.234808 |
| Sykb | NM_011518 | spleen tyrosine kinase | 0.000778 | 2.247891 |
| Ckap2 | NM_001004140 | cytoskeleton associated protein 2 | 0.01118 | 2.248322 |
| Alox5ap | NM_009663 | arachidonate 5-lipoxygenase activating protein | 0.000231 | 2.25004 |
| Ccna2 | NM_009828 | cyclin A2 | 0.023725 | 2.252924 |
| Ccr2 | NM_009915 | chemokine (C-C motif) receptor 2 | 0.017508 | 2.259005 |
| Kcnn4 | NM_008433 | potassium intermediate/small conductance calcium-activated channel, subfamily N, member 4 | 0.001236 | 2.26043 |
| Tm7sf4 | NM_029422 | transmembrane 7 superfamily member 4 | 0.006939 | 2.265429 |
| Chst12 | NM_021528 | carbohydrate sulfotransferase 12 | 0.020102 | 2.277458 |
| Ccl3 | NM_011337 | chemokine (C-C motif) ligand 3 | 0.033032 | 2.289381 |
| Aurkb | NM_011496 | aurora kinase B | 0.007502 | 2.291171 |
| Ctss | NM_021281 | cathepsin S | 3.10E-05 | 2.293827 |
| Pla2g7 | NM_013737 | phospholipase A2, group VII (platelet-activating factor acetylhydrolase, plasma) | 0.001004 | 2.30601 |
| Dok2 | NM_010071 | docking protein 2 | 0.002245 | 2.306102 |
| Il2rg | NM_013563 | interleukin 2 receptor, gamma chain | 8.74E-05 | 2.307857 |
| Klk13 | NM_001039042 | kallikrein related-peptidase 13 | 0.010711 | 2.309787 |
| Atp1a3 | NM_144921 | ATPase, Na+/K+ transporting, alpha 3 polypeptide | 0.002886 | 2.313402 |
| Slc16a3 | NM_030696 | solute carrier family 16 (monocarboxylic acid transporters), member 3 | 0.008852 | 2.318478 |
| Mpeg1 | NM_010821 | macrophage expressed gene 1 | 0.009802 | 2.323121 |
| Igfbp2 | NM_008342 | insulin-like growth factor binding protein 2 | 0.009544 | 2.333119 |
| Scn2b | NM_001014761 | sodium channel, voltage-gated, type II, beta | 5.20E-05 | 2.337565 |
| Islr2 | NM_177193 | immunoglobulin superfamily containing leucine-rich repeat 2 | 0.00215 | 2.338206 |
| H2-DMa | NM_010386 | histocompatibility 2, class II, locus DMa | 0.0005 | 2.345741 |
| Foxp3 | NM_054039 | forkhead box P3 | 0.001701 | 2.34675 |
| Lag3 | NM_008479 | lymphocyte-activation gene 3 | 0.022927 | 2.348245 |
| Ccdc109b | NM_025779 | coiled-coil domain containing 109B | 0.001571 | 2.34892 |
| Neurl3 | NM_153408 | neuralized homolog 3 homolog (Drosophila) | 0.008383 | 2.352709 |
| B4galnt1 | NM_027739 | beta-1,4-N-acetyl-galactosaminyl transferase 1 | 4.53E-05 | 2.368888 |
| Rac2 | NM_009008 | RAS-related C3 botulinum substrate 2 | 7.35E-05 | 2.379268 |
| Fcgr3 | NM_010188 | Fc receptor, IgG, low affinity III | 0.000548 | 2.382594 |
| Cdc20 | NM_023223 | cell division cycle 20 homolog (S. cerevisiae) | 0.016153 | 2.395522 |
| Fcrls | NM_030707 | Fc receptor-like S, scavenger receptor | 0.007415 | 2.404458 |
| C1qtnf6 | NM_028331 | C1q and tumor necrosis factor related protein 6 | 0.001202 | 2.405683 |
| Fst | NM_008046 | follistatin | 0.018796 | 2.411921 |
| Dapp1 | NM_011932 | dual adaptor for phosphotyrosine and 3-phosphoinositides 1 | 0.000175 | 2.413067 |
| Bcl2a1a | NM_009742 | B-cell leukemia/lymphoma 2 related protein A1a | 0.00126 | 2.416858 |
| Ncf2 | NM_010877 | neutrophil cytosolic factor 2 | 0.000705 | 2.417183 |
| Mmp2 | NM_008610 | matrix metallopeptidase 2 | 0.000144 | 2.425422 |
| Cpxm1 | NM_019696 | carboxypeptidase X 1 (M14 family) | 0.000474 | 2.436375 |
| Tnfsf13b | NM_033622 | tumor necrosis factor (ligand) superfamily, member 13b | 0.000267 | 2.449053 |
| Cenpa | NM_007681 | centromere protein A | 0.030377 | 2.473093 |
| Mmp3 | NM_010809 | matrix metallopeptidase 3 | 0.000967 | 2.473696 |
| Col1a1 | NM_007742 | collagen, type I, alpha 1 | 0.000747 | 2.476911 |
| Serpina10 | NM_144834 | serine (or cysteine) peptidase inhibitor, clade A (alpha-1 antiproteinase, antitrypsin), member 10 | 0.005395 | 2.47741 |
| Ncf1 | NM_010876 | neutrophil cytosolic factor 1 | 0.010757 | 2.478851 |
| Ccl2 | NM_011333 | chemokine (C-C motif) ligand 2 | 0.011517 | 2.483152 |
| Sfn | NM_018754 | stratifin | 0.016263 | 2.484865 |
| Apob48r | NM_138310 | apolipoprotein B48 receptor | 0.000247 | 2.49461 |
| Slfn2 | NM_011408 | schlafen 2 | 0.000178 | 2.503062 |
| Gpr34 | NM_011823 | G protein-coupled receptor 34 | 0.002362 | 2.524332 |
| Mfap4 | NM_029568 | microfibrillar-associated protein 4 | 0.001132 | 2.52924 |
| Arntl | NM_007489 | aryl hydrocarbon receptor nuclear translocator-like | 0.049937 | 2.531795 |
| Icos | NM_017480 | inducible T-cell co-stimulator | 0.002828 | 2.548036 |
| Fignl1 | NM_021891 | fidgetin-like 1 | 0.001691 | 2.548983 |
| Ifi27l2a | NM_029803 | interferon, alpha-inducible protein 27 like 2A | 8.02E-06 | 2.561309 |
| H2-DMb1 | NM_010387 | histocompatibility 2, class II, locus Mb1 | 0.002661 | 2.562963 |
| Ly9 | NM_008534 | lymphocyte antigen 9 | 0.000177 | 2.564851 |
| Cd48 | NM_007649 | CD48 antigen | 0.001164 | 2.56864 |
| Socs1 | NM_009896 | suppressor of cytokine signaling 1 | 0.004289 | 2.568723 |
| Tyrobp | NM_011662 | TYRO protein tyrosine kinase binding protein | 0.000902 | 2.580494 |
| H2-DMb2 | NM_010388 | histocompatibility 2, class II, locus Mb2 | 0.000995 | 2.587353 |
| Lcn2 | NM_008491 | lipocalin 2 | 0.01398 | 2.589232 |
| Sfpi1 | NM_011355 | SFFV proviral integration 1 | 0.000447 | 2.592531 |
| Cd300lf | NM_145634 | CD300 antigen like family member F | 0.006165 | 2.595568 |
| Selplg | NM_009151 | selectin, platelet (p-selectin) ligand | 0.003266 | 2.604103 |
| F630028O10Rik | NR_030718 | RIKEN cDNA F630028O10 gene | 0.00633 | 2.617062 |
| Cxcl5 | NM_009141 | chemokine (C-X-C motif) ligand 5 | 0.011509 | 2.617189 |
| Ccdc80 | NM_026439 | coiled-coil domain containing 80 | 0.00143 | 2.625486 |
| Cfp | NM_008823 | complement factor properdin | 0.004166 | 2.630467 |
| Cd27 | NM_001042564 | CD27 antigen | 0.013448 | 2.635738 |
| Mmp9 | NM_013599 | matrix metallopeptidase 9 | 8.93E-05 | 2.637842 |
| Serpina3f | NM_001168295 | serine (or cysteine) peptidase inhibitor, clade A, member 3F | 0.00017 | 2.646283 |
| Mcm6 | NM_008567 | minichromosome maintenance deficient 6 (MIS5 homolog, S. pombe) (S. cerevisiae) | 0.006652 | 2.656434 |
| C6 | NM_016704 | complement component 6 | 0.002101 | 2.65935 |
| Slfn1 | NM_011407 | schlafen 1 | 0.001304 | 2.667767 |
| Syt13 | NM_030725 | synaptotagmin XIII | 0.005038 | 2.668188 |
| Ccr1 | NM_009912 | chemokine (C-C motif) receptor 1 | 0.001904 | 2.668206 |
| Vcam1 | NM_011693 | vascular cell adhesion molecule 1 | 0.018968 | 2.671199 |
| Ctse | NM_007799 | cathepsin E | 0.000332 | 2.689763 |
| Krt19 | NM_008471 | keratin 19 | 0.001326 | 2.698209 |
| Col6a1 | NM_009933 | collagen, type VI, alpha 1 | 0.002531 | 2.701825 |
| Ube2c | NM_026785 | ubiquitin-conjugating enzyme E2C | 0.034129 | 2.704997 |
| Col3a1 | NM_009930 | collagen, type III, alpha 1 | 0.001049 | 2.715243 |
| Ly6d | NM_010742 | lymphocyte antigen 6 complex, locus D | 0.009929 | 2.719923 |
| Cd4 | NM_013488 | CD4 antigen | 0.001908 | 2.727138 |
| Lpxn | NM_134152 | leupaxin | 0.014538 | 2.728547 |
| Itgax | NM_021334 | integrin alpha X | 0.000316 | 2.739886 |
| Efs | NM_010112 | embryonal Fyn-associated substrate | 0.007151 | 2.748649 |
| Ikzf3 | NM_011771 | IKAROS family zinc finger 3 | 4.46E-05 | 2.777094 |
| Ccl9 | NM_011338 | chemokine (C-C motif) ligand 9 | 0.002142 | 2.78313 |
| Serpina3n | NM_009252 | serine (or cysteine) peptidase inhibitor, clade A, member 3N | 0.000235 | 2.787864 |
| Tmsb10 | NM_025284 | thymosin, beta 10 | 0.001661 | 2.789303 |
| Cd247 | NM_031162 | CD247 antigen | 0.005379 | 2.793035 |
| Serpina3h | NM_001034870 | serine (or cysteine) peptidase inhibitor, clade A, member 3H | 1.65E-05 | 2.796579 |
| Cd6 | NM_009852 | CD6 antigen | 1.10E-05 | 2.802885 |
| Gimap7 | NM_146167 | GTPase, IMAP family member 7 | 0.000368 | 2.803307 |
| Cd3e | NM_007648 | CD3 antigen, epsilon polypeptide | 2.45E-05 | 2.815847 |
| Cd3g | NM_009850 | CD3 antigen, gamma polypeptide | 0.000941 | 2.820142 |
| Egr2 | NM_010118 | early growth response 2 | 0.004758 | 2.820361 |
| Cd52 | NM_013706 | CD52 antigen | 0.001237 | 2.820797 |
| Mki67 | NM_001081117 | antigen identified by monoclonal antibody Ki 67 | 0.012089 | 2.823993 |
| Apol7c | NM_175391 | apolipoprotein L 7c | 0.029397 | 2.856421 |
| Snx20 | NM_027840 | sorting nexin 20 | 0.000615 | 2.874568 |
| Gapt | NM_177713 | Grb2-binding adaptor, transmembrane | 0.012272 | 2.884011 |
| Cd2 | NM_013486 | CD2 antigen | 0.000269 | 2.890369 |
| Cdk1 | NM_007659 | cyclin-dependent kinase 1 | 0.010313 | 2.900853 |
| Cdca3 | NM_013538 | cell division cycle associated 3 | 0.01417 | 2.907025 |
| D2Ertd750e | NM_026412 | DNA segment, Chr 2, ERATO Doi 750, expressed | 0.016637 | 2.923318 |
| Mmp12 | NM_008605 | matrix metallopeptidase 12 | 0.001166 | 2.928806 |
| Bcl2a1b | NM_007534 | B-cell leukemia/lymphoma 2 related protein A1b | 7.03E-05 | 2.941827 |
| Cd28 | NM_007642 | CD28 antigen | 0.000168 | 2.955929 |
| Birc5 | NM_009689 | baculoviral IAP repeat-containing 5 | 0.011192 | 2.960667 |
| Ckap2l | NM_181589 | cytoskeleton associated protein 2-like | 0.03076 | 2.961361 |
| Galntl1 | NM_001081421 | UDP-N-acetyl-alpha-D-galactosamine:polypeptide N-acetylgalactosaminyltransferase-like 1 | 0.000178 | 2.964849 |
| Ccl12 | NM_011331 | chemokine (C-C motif) ligand 12 | 0.000186 | 2.966596 |
| Cck | NM_031161 | cholecystokinin | 0.048782 | 2.977462 |
| Igj | NM_152839 | immunoglobulin joining chain | 0.000272 | 2.991706 |
| Cd8a | NM_009857 | CD8 antigen, alpha chain | 0.008096 | 2.992187 |
| Timp1 | NM_011593 | tissue inhibitor of metalloproteinase 1 | 0.012055 | 3.028999 |
| 9830107B12Rik | NM_177824 | RIKEN cDNA 9830107B12 gene | 0.001871 | 3.043993 |
| Lyz2 | NM_017372 | lysozyme 2 | 0.000591 | 3.051281 |
| Gm7455 | NM_001167923 | predicted gene 7455 | 0.012149 | 3.065135 |
| Serpina3g | NM_009251 | serine (or cysteine) peptidase inhibitor, clade A, member 3G | 0.002751 | 3.08286 |
| Ms4a7 | NM_027836 | membrane-spanning 4-domains, subfamily A, member 7 | 0.000846 | 3.087285 |
| S100a4 | NM_011311 | S100 calcium binding protein A4 | 0.006029 | 3.097756 |
| Ctla4 | NM_009843 | cytotoxic T-lymphocyte-associated protein 4 | 0.000113 | 3.110736 |
| Ccl6 | NM_009139 | chemokine (C-C motif) ligand 6 | 0.005508 | 3.245305 |
| Basp1 | NM_027395 | brain abundant, membrane attached signal protein 1 | 0.002237 | 3.314406 |
| Ccl22 | NM_009137 | chemokine (C-C motif) ligand 22 | 0.002299 | 3.314712 |
| Lyz1 | NM_013590 | lysozyme 1 | 0.000268 | 3.335196 |
| Gpnmb | NM_053110 | glycoprotein (transmembrane) nmb | 0.000876 | 3.336071 |
| Ltb | NM_008518 | lymphotoxin B | 0.002615 | 3.357691 |
| H2-M2 | NM_008204 | histocompatibility 2, M region locus 2 | 3.18E-05 | 3.404733 |
| Ltb4r1 | NM_008519 | leukotriene B4 receptor 1 | 0.006639 | 3.461317 |
| Pglyrp1 | NM_009402 | peptidoglycan recognition protein 1 | 0.011954 | 3.480177 |
| Cd3d | NM_013487 | CD3 antigen, delta polypeptide | 0.001743 | 3.493836 |
| Ccr7 | NM_007719 | chemokine (C-C motif) receptor 7 | 0.005281 | 3.518289 |
| Retnla | NM_020509 | resistin like alpha | 0.025015 | 3.571555 |
| Rgs1 | NM_015811 | regulator of G-protein signaling 1 | 6.58E-05 | 3.581174 |
| Ccl17 | NM_011332 | chemokine (C-C motif) ligand 17 | 0.002064 | 3.592335 |
| Ear11 | NM_053113 | eosinophil-associated, ribonuclease A family, member 11 | 0.018106 | 3.697203 |
| Klrg1 | NM_016970 | killer cell lectin-like receptor subfamily G, member 1 | 0.002124 | 3.699619 |
| Cebpe | NM_207131 | CCAAT/enhancer binding protein (C/EBP), epsilon | 0.015221 | 3.714427 |
| F10 | NM_007972 | coagulation factor X | 0.003632 | 3.749046 |
| Pdgfrl | NM_026840 | platelet-derived growth factor receptor-like | 0.008017 | 3.827275 |
| Slc36a2 | NM_153170 | solute carrier family 36 (proton/amino acid symporter), member 2 | 0.00025 | 3.839795 |
| Hp | NM_017370 | haptoglobin | 0.031226 | 3.870553 |
| Galnt6 | NM_172451 | UDP-N-acetyl-alpha-D-galactosamine:polypeptide N-acetylgalactosaminyltransferase 6 | 0.000212 | 3.878774 |
| Art2a | NM_007490 | ADP-ribosyltransferase 2a | 1.11E-05 | 3.896566 |
| Arg1 | NM_007482 | arginase, liver | 0.016169 | 3.918515 |
| Arl11 | NM_177337 | ADP-ribosylation factor-like 11 | 0.000777 | 3.951248 |
| Ccl24 | NM_019577 | chemokine (C-C motif) ligand 24 | 0.008132 | 4.018104 |
| F7 | NM_010172 | coagulation factor VII | 0.009557 | 4.046409 |
| Retnlg | NM_181596 | resistin like gamma | 0.016055 | 4.218991 |
| Ccr4 | NM_009916 | chemokine (C-C motif) receptor 4 | 0.001546 | 4.312705 |
| Mmp7 | NM_010810 | matrix metallopeptidase 7 | 0.000326 | 4.444229 |
| Chi3l3 | NM_009892 | chitinase 3-like 3 | 0.038508 | 4.622626 |
| Ear1 | NM_007894 | eosinophil-associated, ribonuclease A family, member 1 | 2.75E-05 | 4.68219 |
| Pdcd1lg2 | NM_021396 | programmed cell death 1 ligand 2 | 0.00137 | 4.836419 |
| Ear10 | NM_053112 | eosinophil-associated, ribonuclease A family, member 10 | 0.016895 | 4.990777 |
| Lrrn2 | NM_010732 | leucine rich repeat protein 2, neuronal | 0.004386 | 5.459293 |

**Table S3: Genes up-regulated from 4 to 8 weeks and from 8 weeks to 12 weeks**

| Symbol | RefSeq | Gene Name | pvalue.w8_w4 | Log2Rat.w8_w4 | pvalue.w12_w8 | Log2Rat.w12_w8 |
| --- | --- | --- | --- | --- | --- | --- |
| Cdca7l | NM_146040 | cell division cycle associated 7 like | 0.04551 | 0.533737 | 0.041785 | 0.437991 |
| Itpripl2 | NM_001033380 | inositol 1,4,5-triphosphate receptor interacting protein-like 2 | 0.041301 | 0.54802 | 0.048396 | 0.341784 |
| Aldh1a7 | NM_011921 | aldehyde dehydrogenase family 1, subfamily A7 | 0.025895 | 0.574792 | 0.019754 | 1.026329 |
| Bcl6b | NM_007528 | B-cell CLL/lymphoma 6, member B | 0.043134 | 0.594684 | 0.010056 | 1.107977 |
| Rnf150 | NM_177378 | ring finger protein 150 | 0.044605 | 0.600936 | 0.042763 | 0.831038 |
| Lpar1 | NM_172989 | lysophosphatidic acid receptor 1 | 0.018827 | 0.685172 | 0.00362 | 1.369493 |
| Cygb | NM_030206 | cytoglobin | 0.012988 | 0.790019 | 0.038554 | 1.156904 |
| Zcchc24 | NM_001101433 | zinc finger, CCHC domain containing 24 | 0.02635 | 0.791435 | 0.020104 | 0.6807 |
| Rcsd1 | NM_178593 | RCSD domain containing 1 | 0.009416 | 0.79155 | 0.049472 | 0.877275 |
| Pip4k2a | NM_008845 | phosphatidylinositol-5-phosphate 4-kinase, type II, alpha | 0.019249 | 0.843884 | 0.037189 | 0.518859 |
| BC046404 | NM_198861 | cDNA sequence BC046404 | 0.009979 | 0.860633 | 0.023195 | 0.916799 |
| Rtp4 | NM_023386 | receptor transporter protein 4 | 0.005879 | 0.912609 | 0.015459 | 1.065351 |
| Igf1 | NM_184052 | insulin-like growth factor 1 | 0.038754 | 0.921849 | 0.049383 | 0.693516 |
| Hbb-b1 | NM_008220 | hemoglobin, beta adult major chain | 0.040739 | 0.935941 | 0.00103 | 2.469216 |
| Hbb-b2 | NM_016956 | hemoglobin, beta adult minor chain | 0.040543 | 0.938577 | 0.001043 | 2.463061 |
| Mrgpre | NM_175534 | MAS-related GPR, member E | 0.010129 | 0.957408 | 0.021128 | 0.959207 |
| Il33 | NM_133775 | interleukin 33 | 0.02368 | 1.070782 | 0.019279 | 0.72284 |
| Ptger4 | NM_008965 | prostaglandin E receptor 4 (subtype EP4) | 0.037265 | 1.122658 | 0.022757 | 0.805092 |
| Fhl2 | NM_010212 | four and a half LIM domains 2 | 0.015468 | 1.130569 | 0.032237 | 1.18361 |
| Csprs | NM_033616 | component of Sp100-rs | 0.021647 | 1.135435 | 0.019439 | 0.658267 |
| B3galt1 | NM_020283 | UDP-Gal:betaGlcNAc beta 1,3-galactosyltransferase, polypeptide 1 | 0.042175 | 1.162322 | 0.001027 | 1.586701 |
| Kcnf1 | NM_201531 | potassium voltage-gated channel, subfamily F, member 1 | 0.047595 | 1.179628 | 0.001428 | 1.965157 |
| Tpsab1 | NM_031187 | tryptase alpha/beta 1 | 0.027834 | 1.18727 | 0.045166 | 0.816041 |
| Clec4d | NM_010819 | C-type lectin domain family 4, member d | 0.003104 | 1.197652 | 0.024496 | 1.137379 |
| Amica1 | NM_001005421 | adhesion molecule, interacts with CXADR antigen 1 | 0.000446 | 1.271593 | 0.022562 | 0.722403 |
| Tubb2b | NM_023716 | tubulin, beta 2B | 0.019448 | 1.286508 | 0.029876 | 0.75871 |
| Tgfbi | NM_009369 | transforming growth factor, beta induced | 0.008769 | 1.345986 | 0.003879 | 0.78402 |
| Gpr176 | NM_201367 | G protein-coupled receptor 176 | 0.024648 | 1.363133 | 0.03294 | 1.85085 |
| Gng2 | NM_010315 | guanine nucleotide binding protein (G protein), gamma 2 | 0.003563 | 1.370561 | 0.036838 | 0.587516 |
| Isg20 | NM_020583 | interferon-stimulated protein | 0.00569 | 1.380551 | 0.04638 | 0.907485 |
| Plek | NM_019549 | pleckstrin | 0.000717 | 1.422905 | 0.035195 | 0.709655 |
| Stk17b | NM_133810 | serine/threonine kinase 17b (apoptosis-inducing) | 0.009335 | 1.444542 | 0.039301 | 0.711784 |
| Clec4a3 | NM_153197 | C-type lectin domain family 4, member a3 | 0.003768 | 1.481594 | 0.041406 | 0.673553 |
| Tspyl3 | NM_198617 | TSPY-like 3 | 0.027375 | 1.500886 | 0.029634 | 1.664702 |
| Tsks | NM_011651 | testis-specific serine kinase substrate | 0.025608 | 1.539834 | 0.00061 | 1.593501 |
| Marcks | NM_008538 | myristoylated alanine rich protein kinase C substrate | 0.003212 | 1.554741 | 0.029376 | 1.102885 |
| Socs3 | NM_007707 | suppressor of cytokine signaling 3 | 0.015718 | 1.631737 | 0.049423 | 0.443119 |
| E2f1 | NM_007891 | E2F transcription factor 1 | 0.026157 | 1.633328 | 0.009457 | 0.83011 |
| Dclk1 | NM_019978 | doublecortin-like kinase 1 | 0.00278 | 1.644534 | 0.037606 | 0.733285 |
| Svs4 | NM_009300 | seminal vesicle secretory protein 4 | 0.015076 | 1.645145 | 0.045635 | 0.958135 |
| Clec4n | NM_020001 | C-type lectin domain family 4, member n | 0.002318 | 1.674654 | 0.020378 | 0.806206 |
| 2010001M09Rik | NM_027222 | RIKEN cDNA 2010001M09 gene | 0.004346 | 1.676292 | 0.044193 | 2.017606 |
| C5ar1 | NM_007577 | complement component 5a receptor 1 | 0.003796 | 1.739229 | 0.017323 | 1.092583 |
| Cd68 | NM_009853 | CD68 antigen | 0.000527 | 1.819702 | 0.027821 | 1.227413 |
| Fgl2 | NM_008013 | fibrinogen-like protein 2 | 0.001127 | 1.845228 | 0.028452 | 0.91238 |
| Cd300lb | NM_199221 | CD300 antigen like family member B | 0.000365 | 1.973003 | 0.009164 | 2.398871 |
| Mrc1 | NM_008625 | mannose receptor, C type 1 | 0.029252 | 1.984891 | 0.018543 | 1.068554 |
| Pf4 | NM_019932 | platelet factor 4 | 0.017755 | 2.027711 | 0.01224 | 1.582629 |
| Lax1 | NM_172842 | lymphocyte transmembrane adaptor 1 | 0.032565 | 2.040322 | 0.046616 | 1.568796 |
| Cxcr4 | NM_009911 | chemokine (C-X-C motif) receptor 4 | 0.008241 | 2.052685 | 0.010787 | 1.440319 |
| Il1rl1 | NM_010743 | interleukin 1 receptor-like 1 | 0.014965 | 2.053573 | 0.045629 | 1.355163 |
| Cd53 | NM_007651 | CD53 antigen | 0.002417 | 2.073147 | 0.005519 | 0.952529 |
| Alas2 | NM_009653 | aminolevulinic acid synthase 2, erythroid | 0.000726 | 2.109231 | 0.030471 | 1.441122 |
| Cd79b | NM_008339 | CD79B antigen | 0.001875 | 2.174497 | 0.037507 | 1.737737 |
| Hba-a2 | NM_001083955 | hemoglobin alpha, adult chain 2 | 0.00666 | 2.186738 | 0.047267 | 0.986712 |
| S100a9 | NM_009114 | S100 calcium binding protein A9 (calgranulin B) | 0.00389 | 2.197376 | 0.002471 | 1.759036 |
| Lox | NM_010728 | lysyl oxidase | 0.007999 | 2.314733 | 0.038004 | 0.739722 |
| S100a8 | NM_013650 | S100 calcium binding protein A8 (calgranulin A) | 0.001166 | 2.587286 | 0.034825 | 0.810592 |
| Pou2af1 | NM_011136 | POU domain, class 2, associating factor 1 | 0.013795 | 2.688214 | 0.012 | 1.634275 |
| Il7r | NM_008372 | interleukin 7 receptor | 0.002897 | 2.689856 | 0.018449 | 1.120188 |
| Ttll2 | NM_001098267 | tubulin tyrosine ligase-like family, member 2 | 0.010189 | 2.787324 | 0.008236 | 2.718184 |
| Slamf6 | NM_030710 | SLAM family member 6 | 6.17E-05 | 2.790791 | 0.000831 | 1.645037 |
| P2ry10 | NM_172435 | purinergic receptor P2Y, G-protein coupled 10 | 0.000578 | 2.873697 | 0.00503 | 1.508612 |
| Ccl8 | NM_021443 | chemokine (C-C motif) ligand 8 | 0.001235 | 3.351744 | 0.003599 | 4.964852 |
| Tigit | NM_001146325 | T cell immunoreceptor with Ig and ITIM domains | 0.01051 | 3.724318 | 0.009439 | 1.105307 |
| Pla2g2d | NM_011109 | phospholipase A2, group IID | 0.037531 | 4.184986 | 0.014708 | 1.430626 |

**Table S4: Genes up-regulated from 4 to 8 weeks but down-regulated from 8 weeks to 12 weeks**

| Symbol | RefSeq | Gene Name | pvalue.w8_w4 | Log2Rat.w8_w4 | pvalue.w12_w8 | Log2Rat.w12_w8 |
| --- | --- | --- | --- | --- | --- | --- |
| Ankrd55 | NM_029898 | ankyrin repeat domain 55 | 0.044745 | 0.455022 | 0.024654 | -0.65869 |
| F5 | NM_007976 | coagulation factor V | 0.027065 | 0.735844 | 0.045629 | -0.93706 |
| Naaladl1 | NM_001009546 | N-acetylated alpha-linked acidic dipeptidase-like 1 | 0.021853 | 0.846529 | 0.02427 | -0.86642 |
| Gbp10 | NM_001039646 | guanylate-binding protein 10 | 0.006976 | 0.989023 | 0.008067 | -1.12024 |
| Trfr2 | NM_015799 | transferrin receptor 2 | 0.032893 | 1.043818 | 0.027622 | -1.07725 |
| Hal | NM_010401 | histidine ammonia lyase | 0.005582 | 1.158314 | 0.018212 | -0.94607 |
| Slc26a6 | NM_134420 | solute carrier family 26, member 6 | 0.015459 | 1.228326 | 0.044917 | -0.76151 |
| Tgtp | NM_011579 | T-cell specific GTPase 1 | 0.032777 | 1.240683 | 0.048558 | -0.69531 |
| Tlr12 | NM_205823 | toll-like receptor 12 | 0.004228 | 1.513596 | 0.04844 | -0.8008 |
| Ltbp2 | NM_013589 | latent transforming growth factor beta binding protein 2 | 0.000807 | 1.559864 | 0.039711 | -0.65179 |
| Naip5 | NM_010870 | NLR family, apoptosis inhibitory protein 5 | 0.00029 | 1.707776 | 0.032253 | -1.0931 |
| Pira3 | NR_028115 | leukocyte immunoglobulin-like receptor, subfamily A (with TM domain), member 6 | 0.020936 | 1.74164 | 0.044796 | -0.86078 |
| Il4i1 | NM_010215 | interleukin 4 induced 1 | 0.005334 | 1.75771 | 0.046159 | -0.71969 |
| Tnfrsf9 | NM_011612 | tumor necrosis factor receptor superfamily, member 9 | 0.000851 | 1.766566 | 0.013217 | -0.78408 |
| Mpa2l | NM_194336 | macrophage activation 2 like | 0.013549 | 1.77355 | 0.018869 | -1.0629 |
| Gbp1 | NM_010259 | guanylate binding protein 1 | 0.008013 | 2.004004 | 0.030949 | -1.2911 |
| Gbp8 | NM_029509 | guanylate-binding protein 8 | 0.000493 | 2.015691 | 0.009206 | -1.60703 |
| Cfi | NM_007686 | complement component factor i | 0.002577 | 2.046374 | 0.033524 | -0.78024 |
| Evi2b | NM_146023 | ecotropic viral integration site 2b | 0.000254 | 2.093729 | 0.049936 | -0.51634 |
| Plk1 | NM_011121 | polo-like kinase 1 (Drosophila) | 0.025722 | 2.42698 | 0.02813 | -0.62363 |
| Btnl2 | NM_079835 | butyrophilin-like 2 | 0.000347 | 2.523709 | 0.002651 | -1.0395 |
| Cdc6 | NM_011799 | cell division cycle 6 homolog (S. cerevisiae) | 0.001056 | 2.712363 | 0.001945 | -1.01357 |
| A530064D06Rik | NM_178796 | RIKEN cDNA A530064D06 gene | 0.002521 | 3.220131 | 0.011556 | -1.34012 |
| 2810417H13Rik | NM_026515 | RIKEN cDNA 2810417H13 gene | 0.00397 | 3.563131 | 0.026461 | -0.84421 |
| Chi3l4 | NM_145126 | chitinase 3-like 4 | 0.006312 | 4.103208 | 0.021903 | -2.1802 |

**Table S5: Genes down-regulated from 4 to 8 weeks but up-regulated from 8 weeks to 12 weeks**

| Symbol | RefSeq | Gene Name | pvalue.w8_w4 | Log2Rat.w8_w4 | pvalue.w12_w8 | Log2Rat.w12_w8 |
| --- | --- | --- | --- | --- | --- | --- |
| Hapln1 | NM_013500 | hyaluronan and proteoglycan link protein 1 | 0.000809 | -2.04009 | 0.018646 | 1.006667 |
| Cyp2d9 | NM_010006 | cytochrome P450, family 2, subfamily d, polypeptide 9 | 0.012045 | -2.01929 | 0.043473 | 1.97382 |
| Mpv17l | NM_033564 | Mpv17 transgene, kidney disease mutant-like | 0.018491 | -1.52019 | 0.018172 | 1.423119 |
| Spag16 | NM_029160 | sperm associated antigen 16 | 0.045838 | -1.36413 | 0.047637 | 1.410503 |
| Crem | NM_013498 | cAMP responsive element modulator | 0.012498 | -1.01281 | 0.005035 | 0.936152 |
| Ctxn3 | NM_001134697 | cortexin 3 | 0.03462 | -0.97074 | 0.006978 | 2.600901 |
| Higd1c | NM_001002900 | HIG1 domain family, member 1C | 0.020578 | -0.91985 | 0.01308 | 1.515427 |
| Ly6f | NM_008530 | lymphocyte antigen 6 complex, locus F | 0.013231 | -0.91297 | 0.025569 | 2.140476 |
| Car5a | NM_007608 | carbonic anhydrase 5a, mitochondrial | 0.008437 | -0.88783 | 0.014197 | 1.426361 |
| Adrb2 | NM_007420 | adrenergic receptor, beta 2 | 0.018642 | -0.76837 | 0.016446 | 0.725741 |
| Ism1 | NM_001126490 | isthmin 1 homolog (zebrafish) | 0.040403 | -0.58347 | 0.02404 | 0.699974 |
| 2310028H24Rik | NM_027993 | RIKEN cDNA 2310028H24 gene | 0.03845 | -0.54494 | 0.018106 | 0.632322 |
| BC031441 | NM_146249 | cDNA sequence BC031441 | 0.049119 | -0.41883 | 0.004355 | 0.885131 |
| BC026439 | NM_172378 | cDNA sequence BC026439 | 0.044041 | -0.94115 | 0.023541 | -1.0868 |

**Table S6: Genes no changes from 4 to 8 weeks but down-regulated from 8 weeks to 12 weeks**

| Symbol | RefSeq | Gene Name | pvalue.w12_w8 | Log2Rat.w12_w8 |
| --- | --- | --- | --- | --- |
| Cml3 | NM_053097 | camello-like 3 | 0.00504 | -2.02104 |
| Neurog2 | NM_009718 | neurogenin 2 | 0.000118 | -1.99432 |
| Cnga2 | NM_007724 | cyclic nucleotide gated channel alpha 2 | 0.013025 | -1.95296 |
| Gm884 | NM_001033434 | predicted gene 884 | 0.025293 | -1.8217 |
| Alpk2 | NM_001037294 | alpha-kinase 2 | 0.03793 | -1.77589 |
| Flrt1 | NM_201411 | fibronectin leucine rich transmembrane protein 1 | 0.046161 | -1.71672 |
| Cyp7b1 | NM_007825 | cytochrome P450, family 7, subfamily b, polypeptide 1 | 0.016067 | -1.70993 |
| Prkar2b | NM_011158 | protein kinase, cAMP dependent regulatory, type II beta | 0.011922 | -1.66404 |
| Clec4a4 | NM_001005860 | C-type lectin domain family 4, member a4 | 0.012917 | -1.65843 |
| E2f8 | NM_001013368 | E2F transcription factor 8 | 0.020771 | -1.65754 |
| Il22ra1 | NM_178257 | interleukin 22 receptor, alpha 1 | 0.021753 | -1.64929 |
| Hrg | NM_053176 | histidine-rich glycoprotein | 0.015553 | -1.62341 |
| 1700001C02Rik | NM_029285 | RIKEN cDNA 1700001C02 gene | 0.030798 | -1.60507 |
| Dnase1 | NM_010061 | deoxyribonuclease I | 0.019752 | -1.55962 |
| Airn | NR_027784 | antisense Igf2r RNA | 0.000946 | -1.4719 |
| Pbx4 | NM_001024954 | pre-B-cell leukemia homeobox 4 | 0.028732 | -1.47043 |
| AI747699 | NM_001013770 | expressed sequence AI747699 | 0.028882 | -1.46827 |
| Rims1 | NM_183018 | regulating synaptic membrane exocytosis 1 | 0.046092 | -1.45443 |
| Clec2h | NM_053165 | C-type lectin domain family 2, member h | 0.010062 | -1.30755 |
| Adam26b | NM_001009547 | a disintegrin and metallopeptidase domain 26B | 0.027127 | -1.3006 |
| Pigr | NM_011082 | polymeric immunoglobulin receptor | 0.000584 | -1.29522 |
| Trim63 | NM_001039048 | tripartite motif-containing 63 | 0.013896 | -1.2892 |
| Acsm3 | NM_212442 | acyl-CoA synthetase medium-chain family member 3 | 0.003907 | -1.24343 |
| Upk3a | NM_023478 | uroplakin 3A | 0.048708 | -1.22834 |
| Ngfr | NM_033217 | nerve growth factor receptor (TNFR superfamily, member 16) | 0.02522 | -1.21091 |
| Kirrel2 | NM_172898 | kin of IRRE like 2 (Drosophila) | 0.003037 | -1.19298 |
| Clic6 | NM_172469 | chloride intracellular channel 6 | 0.046352 | -1.18582 |
| Clic3 | NM_027085 | chloride intracellular channel 3 | 0.026206 | -1.15528 |
| Car4 | NM_007607 | carbonic anhydrase 4 | 0.036612 | -1.14674 |
| Ubxn10 | NM_178671 | UBX domain protein 10 | 0.017207 | -1.14067 |
| A230065H16Rik | NM_001101503 | RIKEN cDNA A230065H16 gene | 0.003408 | -1.13428 |
| Ugt2b38 | NM_133894 | UDP glucuronosyltransferase 2 family, polypeptide B38 | 0.046728 | -1.12679 |
| Tmem130 | NM_177735 | transmembrane protein 130 | 0.009076 | -1.11806 |
| Itih1 | NM_008406 | inter-alpha trypsin inhibitor, heavy chain 1 | 0.00579 | -1.1138 |
| Slc13a2 | NM_022411 | solute carrier family 13 (sodium-dependent dicarboxylate transporter), member 2 | 0.01983 | -1.10907 |
| Rtp3 | NM_153100 | receptor transporter protein 3 | 0.008325 | -1.10443 |
| Atp10b | NM_176999 | ATPase, class V, type 10B | 0.027967 | -1.09974 |
| Mug-ps1 | NR_027619 | murinoglobulin, pseudogene 1 | 0.02913 | -1.09254 |
| Nepn | NM_025684 | nephrocan | 0.007235 | -1.0449 |
| Slc7a13 | NM_028746 | solute carrier family 7, (cationic amino acid transporter, y+ system) member 13 | 0.010341 | -1.04089 |
| Fam151a | NM_146149 | family with sequence simliarity 151, member A | 0.002982 | -1.03338 |
| Slc1a2 | NM_011393 | solute carrier family 1 (glial high affinity glutamate transporter), member 2 | 0.047317 | -1.00254 |

**Table S7: Genes no changes from 4 to 8 weeks but up-regulated from 8 weeks to 12 weeks**

| Symbol | RefSeq | Gene Name | pvalue.w12_w8 | Log2Rat.w12_w8 |
| --- | --- | --- | --- | --- |
| Gpsm1 | NM_153410 | G-protein signalling modulator 1 (AGS3-like, C. elegans) | 0.007091 | 1.000061 |
| Cox7b2 | NM_030052 | cytochrome c oxidase subunit VIIb2 | 0.007288 | 1.001076 |
| Fkbp5 | NM_010220 | FK506 binding protein 5 | 0.018627 | 1.004153 |
| AI506816 | NR_015554 | expressed sequence AI506816 | 0.034319 | 1.021001 |
| Ggnbp1 | NM_027544 | gametogenetin binding protein 1 | 0.043738 | 1.023624 |
| Per1 | NM_011065 | period homolog 1 (Drosophila) | 0.010877 | 1.038799 |
| Rftn2 | NM_028713 | raftlin family member 2 | 0.033425 | 1.043204 |
| Cdo1 | NM_033037 | cysteine dioxygenase 1, cytosolic | 0.035208 | 1.051682 |
| Cdr2 | NM_007672 | cerebellar degeneration-related 2 | 0.013519 | 1.063899 |
| Rbm3 | NM_016809 | RNA binding motif protein 3 | 0.034744 | 1.078171 |
| Ptch2 | NM_008958 | patched homolog 2 | 0.017875 | 1.079378 |
| 2700023E23Rik | NR_015531 | RIKEN cDNA 2700023E23 gene | 0.025218 | 1.093322 |
| Apold1 | NM_001109914 | apolipoprotein L domain containing 1 | 0.036985 | 1.098033 |
| Lilra5 | NM_001081239 | leukocyte immunoglobulin-like receptor, subfamily A (with TM domain), member 5 | 0.039288 | 1.11535 |
| Steap1 | NM_027399 | six transmembrane epithelial antigen of the prostate 1 | 0.017763 | 1.122905 |
| Tgfb3 | NM_009368 | transforming growth factor, beta 3 | 0.018366 | 1.124857 |
| BC024479 | NM_146222 | cDNA sequence BC024479 | 0.030452 | 1.132452 |
| Slc24a3 | NM_053195 | solute carrier family 24 (sodium/potassium/calcium exchanger), member 3 | 0.028725 | 1.146963 |
| Xbp1 | NM_013842 | X-box binding protein 1 | 0.026275 | 1.15922 |
| Gata6 | NM_010258 | GATA binding protein 6 | 0.031035 | 1.160793 |
| Krt23 | NM_033373 | keratin 23 | 0.023588 | 1.16172 |
| Abca8a | NM_153145 | ATP-binding cassette, sub-family A (ABC1), member 8a | 0.00379 | 1.187944 |
| Gt(ROSA)26Sor | NR_027010 | gene trap ROSA 26, Philippe Soriano | 0.013475 | 1.196292 |
| Islr | NM_012043 | immunoglobulin superfamily containing leucine-rich repeat | 0.02391 | 1.197657 |
| Slco1a6 | NM_023718 | solute carrier organic anion transporter family, member 1a6 | 0.044783 | 1.238849 |
| Vgll3 | NM_028572 | vestigial like 3 (Drosophila) | 0.011006 | 1.283384 |
| Ngf | NM_013609 | nerve growth factor | 0.003591 | 1.288599 |
| Rerg | NM_181988 | RAS-like, estrogen-regulated, growth-inhibitor | 0.027432 | 1.289342 |
| Tmem204 | NM_001001183 | transmembrane protein 204 | 0.045061 | 1.298845 |
| C630043F03Rik | NR_027923 | RIKEN cDNA C630043F03 gene | 0.026954 | 1.309805 |
| Ctgf | NM_010217 | connective tissue growth factor | 0.020331 | 1.311816 |
| LOC554292 | NM_001024672 | Mettl7a2-Higd1c readthrough transcript | 0.044972 | 1.313153 |
| Tmprss6 | NM_027902 | transmembrane serine protease 6 | 0.031312 | 1.423723 |
| Ms4a6c | NM_028595 | membrane-spanning 4-domains, subfamily A, member 6C | 0.00219 | 1.455228 |
| Ebf3 | NM_010096 | early B-cell factor 3 | 0.013891 | 1.466752 |
| Tlr7 | NM_133211 | toll-like receptor 7 | 0.003602 | 1.50339 |
| 9430020K01Rik | NM_001081963 | RIKEN cDNA 9430020K01 gene | 0.005061 | 1.512766 |
| Loxl1 | NM_010729 | lysyl oxidase-like 1 | 0.015559 | 1.541852 |
| Gm5640 | NM_001099302 | predicted gene 5640 | 0.007276 | 1.542545 |
| Kng1 | NM_023125 | kininogen 1 | 0.014449 | 1.545446 |
| Gpr183 | NM_183031 | G protein-coupled receptor 183 | 0.00718 | 1.582884 |
| Fbln5 | NM_011812 | fibulin 5 | 0.044587 | 1.645956 |
| Ly6c2 | NM_001099217 | lymphocyte antigen 6 complex, locus C2 | 0.006784 | 1.648515 |
| Slc10a6 | NM_029415 | solute carrier family 10 (sodium/bile acid cotransporter family), member 6 | 0.005798 | 1.665904 |
| Prrx1 | NM_175686 | paired related homeobox 1 | 0.01017 | 1.667472 |
| Tpsb2 | NM_010781 | tryptase beta 2 | 0.004797 | 1.679666 |
| Ogn | NM_008760 | osteoglycin | 0.008794 | 1.696881 |
| Tpst1 | NM_013837 | protein-tyrosine sulfotransferase 1 | 0.032052 | 1.712363 |
| A130040M12Rik | NR_002860 | RIKEN cDNA A130040M12 gene | 0.021732 | 1.769425 |
| Akap12 | NM_031185 | A kinase (PRKA) anchor protein (gravin) 12 | 0.001969 | 1.802491 |
| Kcnj8 | NM_008428 | potassium inwardly-rectifying channel, subfamily J, member 8 | 0.008238 | 1.872222 |
| Colec11 | NM_027866 | collectin sub-family member 11 | 0.047417 | 1.890899 |
| Grem1 | NM_011824 | gremlin 1 | 0.033643 | 1.896251 |
| A530016L24Rik | NM_177039 | RIKEN cDNA A530016L24 gene | 0.00821 | 1.926771 |
| Fxyd6 | NM_022004 | FXYD domain-containing ion transport regulator 6 | 0.013882 | 1.932467 |
| Gcm1 | NM_008103 | glial cells missing homolog 1 (Drosophila) | 0.024902 | 1.938603 |
| Lyve1 | NM_053247 | lymphatic vessel endothelial hyaluronan receptor 1 | 0.007574 | 2.059639 |
| Ccl28 | NM_020279 | chemokine (C-C motif) ligand 28 | 0.026432 | 2.493383 |
| Dcn | NM_007833 | decorin | 0.014596 | 2.514415 |
| Scd1 | NM_009127 | stearoyl-Coenzyme A desaturase 1 | 0.020361 | 2.554422 |
| Slpi | NM_011414 | secretory leukocyte peptidase inhibitor | 0.003989 | 3.193522 |
| Igf2 | NM_010514 | insulin-like growth factor 2 | 0.043388 | 3.735341 |
| Cox8b | NM_007751 | cytochrome c oxidase, subunit VIIIb | 0.03443 | 4.135582 |
